# Neural probe system for behavioral neuropharmacology by bi-directional wireless drug delivery and electrophysiology in socially interacting mice

**DOI:** 10.1038/s41467-022-33296-8

**Published:** 2022-09-21

**Authors:** Yousang Yoon, Hyogeun Shin, Donghak Byun, Jiwan Woo, Yakdol Cho, Nakwon Choi, Il-Joo Cho

**Affiliations:** 1 Brain Science Institute, Korea Institute of Science and Technology (KIST), Seoul, 02792 Republic of Korea; 2 Research Animal Resource Center, Korea Institute of Science and Technology (KIST), Seoul, 02792 Republic of Korea; 3 KU-KIST Graduate School of Converging Science and Technology, Korea University, Seoul, 02841 Republic of Korea; 4 Department of Biomedical Sciences, College of Medicine, Korea University, Seoul, 02841 Republic of Korea

**Keywords:** Extracellular recording, Drug delivery, Motor control, Social behaviour

## Abstract

Assessing the neurological and behavioral effects of drugs is important in developing pharmacological treatments, as well as understanding the mechanisms associated with neurological disorders. Herein, we present a miniaturized, wireless neural probe system with the capability of delivering drugs for the real-time investigation of the effects of the drugs on both behavioral and neural activities in socially interacting mice. We demonstrate wireless drug delivery and simultaneous monitoring of the resulting neural, behavioral changes, as well as the dose-dependent and repeatable responses to drugs. Furthermore, in pairs of mice, we use a food competition assay in which social interaction was modulated by the delivery of the drug, and the resulting changes in their neural activities are analyzed. During modulated food competition by drug injection, we observe changes in neural activity in mPFC region of a participating mouse over time. Our system may provide new opportunities for the development of studying the effects of drugs on behaviour and neural activity.

## Abstract (web summary)

Technologies for monitoring electrophysiological effects of drugs in behaving animals have limitations. Here the authors report a wireless neural probe system with drug delivery capability for real-time monitoring of drug effects.

## Introduction

Along with extensive studies to determine the mechanisms of various brain diseases, much attention has been directed to the development of drugs for pharmacological treatment^1,2^. However, despite the recent studies that identified the neural circuits that underlie the pathophysiology of brain disease, there has been limited evaluation of the efficacy of drugs to manipulate neural circuits because we do not have an effective system that allows the in-depth determination of their pharmacological effect on neural circuits and behaviors^3^. A typical method used in neuropharmacology is a neural implant that delivers drugs directly into the local region of the brain. By utilizing microfabrication techniques, the neural implants have been miniaturized, allowing precise delivery of drugs while minimizing damage to and inflammation of the brain. In addition, the implants allowed the integration of the microelectrodes that monitored the real-time changes in neural activities induced by the drugs^4–10^. This allowed in-depth monitoring of the effects of the drugs, and it made them promising tools for use in neuropharmacology. However, almost all neural implants required an external pump for the delivery of the drug, so the animals must be anesthetized or tethered during the experiments. Unfortunately, due to the tether, these methods disturb the animals’ behaviors, and, even worse, they increase stress and anxiety in the animals, the effects of which are difficult to distinguish from the effects of the drug^11,12^. In addition, the tethered operation restricts the application to social studies using multiple animals. Therefore, for the accurate evaluation of the neuropharmacological effects even applicable to social interactions, the development of a pump-integrated wireless neural probe system is strongly desired.

The miniaturized neural probe system that was developed recently allows the wireless delivery of drugs into the brains of freely behaving animals by connecting a commercially available wireless pump with a neural implant^13^. However, the system, including the wireless pump, is too bulky to be implanted in small animals, such as mice, which are the most widely utilized animal in neuroscience research^14^. Besides, the system does not have wireless neural recording capability, which limits the study of the correlation between the behavioral and neural signal changes induced by the drug. Recently, another group reported the use of wireless, miniaturized neural implants that can deliver finite volumes of drugs while simultaneously performing optogenetics, demonstrating chronic dual-mode neuromodulation^15,16^ (Supplementary Table 1). However, there are unmet needs relative to monitoring the change of neural activity and controlling the dosages of drugs. It is essential to meet these needs in order to investigate the chronic effects of the drugs depending on the dose as well as to investigate the correlation between the changes in the behavioral and neural activities induced by the drugs. In addition, the effects of drugs on a group of freely interacting animals have not been demonstrated to date, and this research must be done to enable the investigation of the individual and social impacts of the drugs on neuropsychiatric diseases. To develop a neural probe system for the neuropharmacological investigation of neural circuits in vivo, the probe system should be (1) integrated with a miniaturized pump that enables the control of dosages and consistently reliable delivery of drugs, (2) capable of observing neural and behavioral effects simultaneously with the delivery of drugs, and (3) a wireless system that enables the investigation of pharmacological effects on multiple, socially interacting animals.

Herein, we present a miniaturized wireless neural probe system that is capable of chronic, dose-controllable drug delivery and simultaneous monitoring of the neural signal and behavioral changes. We monolithically integrated a neural probe, a miniaturized electrolytic pump, and a bi-directional wireless communication module in a single platform. The miniaturized electrolytic pump enables the infusion of a precise amount of the drug into the local region of the brain, and it allows control of the dosages, reliable injection, refillable reservoirs, and low power consumption. The bi-directional wireless communication module allows both control of drug infusion and simultaneous transmission of recorded neural signals from multiple animals while monitoring behavioral changes. By fully utilizing the abilities of our neural probe system, we demonstrate (1) wireless drug delivery and simultaneous monitoring of neural, behavioral changes, (2) controllable, chronic drug delivery and monitoring of neural, behavioral changes, and (3) drug-induced modulation of social interaction in freely behaving mice, and monitoring of the resulting neural changes. Especially in a food competition assay, we show how the drug affects the competition pattern and changes the neural activity of the brain region related to social behaviors over time, which were demonstrated by correlating with the changes in their neural activities. To the best of our knowledge, this is an unprecedented demonstration that is only possible through wireless drug delivery along with the simultaneous, real-time monitoring of behavior and electrophysiology. Our wireless neural probe system has versatile abilities, and we expect that it can be applied immediately to behavioral neuropharmacology and contribute to the development of pharmacotherapy for various brain diseases.

## Results

### Design of the wireless neural probe system integrated with a miniaturized pump

**Fig. 1:**
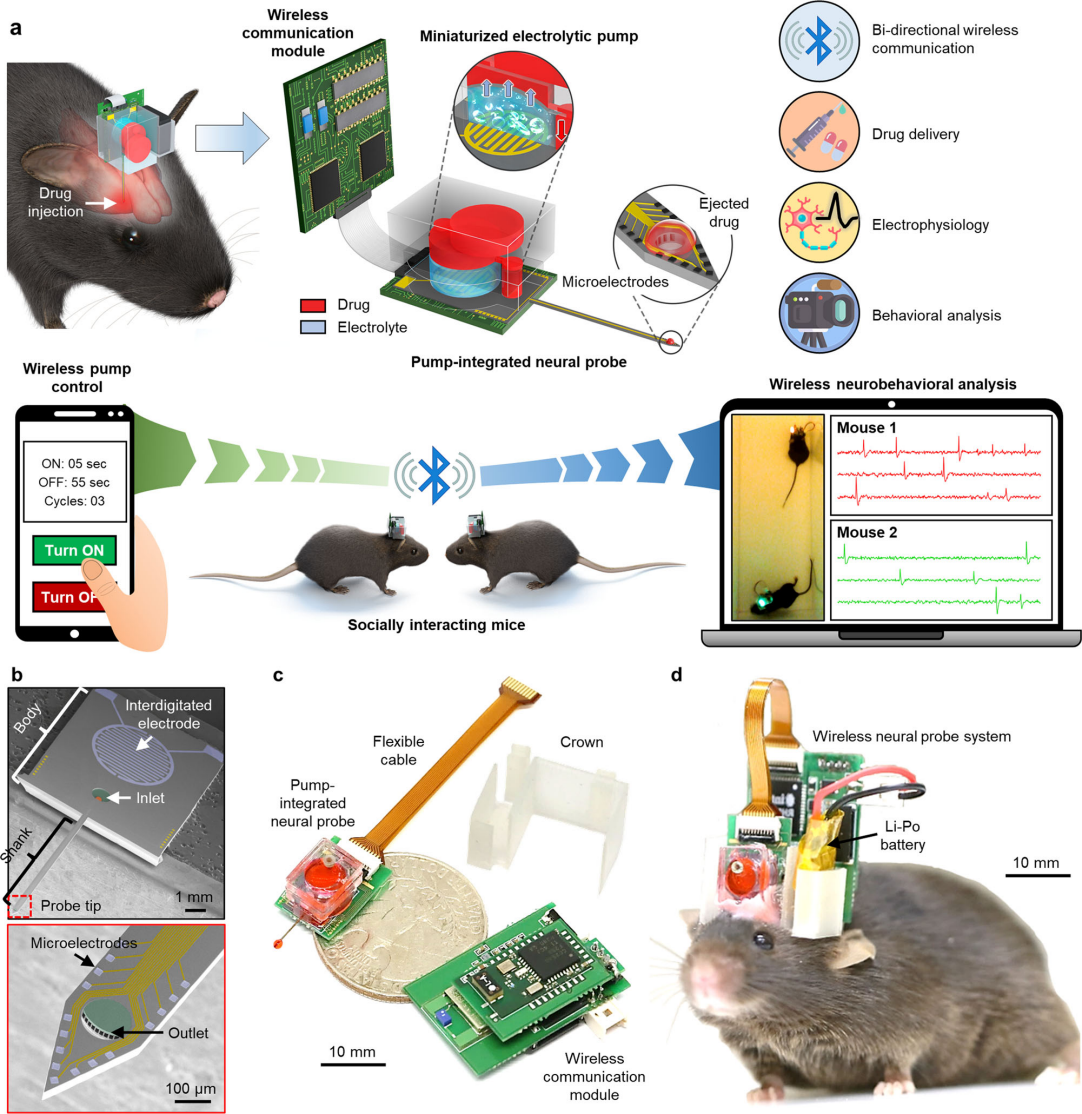
Design and features of the wireless neural probe system with drug delivery capability. **a** Schematic illustrations of the wireless neural probe system and its capabilities for behavioral neuropharmacology. **b** Scanning electron microscope (SEM) image of the neural probe showing the interdigitated electrode (IDE) and the fluid inlet. The magnified SEM image showing a probe shank tip, including 16 microelectrodes and microfluidic channels. **c** Photograph showing the overall configuration of the system. **d** Photograph showing the wireless neural probe system mounted on the head of a mouse.

The wireless neural probe system integrated with a miniaturized pump was implemented by integrating three subsystems in a single platform, i.e., (1) a neural probe embedded with microfluidic channels for the delivery of drugs and an electrode array for recording neural signals; (2) a miniaturized electrolytic pump that generates pneumatic pressure by electrolyzing the electrolyte for drug infusion; and (3) a wireless communication module that allows a user to wirelessly control the pump while simultaneously monitoring the neural activities recorded by the microelectrode array in real time (Fig. 1a). The neural probe was fabricated based on our previously reported method^5,6^ with modifications on the design to integrate the electrolytic pump. The neural probe largely can be divided into a body and a shank (Fig. 1b). An interdigitated electrode (IDE) for performing electrolysis and a drug inlet are located on the body of the probe. Sixteen microelectrodes, microfluidic channels, and a drug outlet are integrated on the shank of the probe (Fig. 1b). Also, the microelectrodes were electroplated with Pt black to enhance the neural recording performance^17–21^. The long-term stability of the probe, including Pt black microelectrodes, was demonstrated in our previously reported study^21^. In addition, the microfluidic channels were designed to have low fluidic resistance, which enables the delivery of the drug by low pressure that is generated from a miniaturized, electrolytic pump. To achieve low fluidic resistance, we maximized the number of channels to ten and increased the dimensions of the channel while maintaining the structure of the small probe (width = 10 μm; length = 7400 μm; height = 12 μm per channel). This resulted in a fluidic resistance that was four times lower than our previously reported microfluidic channels embedded in the neural probe. Thus, the width, thickness, and length of the probe shank we fabricated were 0.24, 0.04, and 6 mm, respectively (the detailed dimensions and materials of the probe were provided in Supplementary Figs. 1 and 2). The small cross-sectional area of the probe minimizes the damage to the tissue during the insertion into a brain as well as the chronic immune response in the brain^7^. Also, its length is sufficient to access all regions of the brain of a mouse.

The dimensions of the miniaturized electrolytic pump (width = 7 mm, length = 9 mm, and height = 6 mm) were attained by stacking three layers of polydimethylsiloxane (PDMS)-based reservoirs, including the fluidic channels, on the IDE of the body of the neural probe. The bottom reservoir contained an electrolyte (40 μl) in the bottom layer, the middle reservoir contained a drug (25 μl), and the top reservoir contained the drug that was used to refill the middle reservoir (25 μl). To infuse the drug to the outlet of the neural probe, voltage is applied to the IDE to initiate electrolysis in the bottom layer, which generates pneumatic pressure and pushes the drug in the middle layer to the outlet of the probe through the microfluidic channels (Fig. 1a). The generation of pneumatic pressure for drug infusion by electrolysis allows low power consumption and low-voltage operation without any degradation of the properties of the drug due to the generation of heat^22^. In addition, the electrolytic pump provides dose-controllable and chronically reliable delivery of the drug, which allows the observation of dose-dependent effects and the long-term effects of the drug.

The wireless communication system is implemented with a commercial electrophysiology chip for recording and amplifying the neural signals, a microcontroller unit (MCU) for data processing, and a bi-directional Bluetooth low energy (BLE) module to control the electrolytic pump and transmit neural signals wirelessly. The BLE module provides the selection of the target animal among a crowded group as well as persistent connection even with a freely moving subject. The data processing unit (i.e., MCU) converts a large amount of analog signals recorded from the sixteen electrodes of the neural probe to only neural spikes with timing information. This allows wireless transmission of neural signals even with the low data rates (<1 Mbps) of BLE, which enables monitoring of neural activities from 16 electrodes in real time.

The neural probe integrated with an electrolytic pump (0.7 g), the wireless communication system (2.36 g), and the 30 mAh Li-Po battery (0.94 g) were mounted on the head of a mouse. The components were connected electrically by flexible cables and packaged in a 3D-printed crown (0.6 g) (Fig. 1c, d). The highly packed integration of multiple modalities (i.e., drug delivery, electrophysiology, and wireless communication) in a single platform enabled the entire system to be miniaturized (~2 cm^3^) and have a light weight of only 4.6 g. When the system’s weight exceeds 10% of the mice’s bodyweight, it is known that their behavior could be affected^23^. However, after a week of recovery and adaptation to the system, we observed that there was little difference in the distance traveled by the mice before and after mounting the system (4.6 g) in the open-field test (OFT) for 30 min. Thus, we confirmed that the weight of the system did not nearly interfere with the behavior of the mice (Supplementary Figs. 3 and 4 and Supplementary Movie 1). Also, through the successive OFTs with normal mice that were not mounted with the system (Supplementary Fig. 5), we found that the average value of distance traveled was slightly decreased even though the difference was not statistically meaningful. We inferred that the slight decrease after mounting the system was due to the normal habituation to a novel environment (i.e., an open box). Thus, our system provides drug delivery and electrophysiology wirelessly in freely behaving small animals.

### Design and operation principle of the miniaturized electrolytic pump

**Fig. 2:**
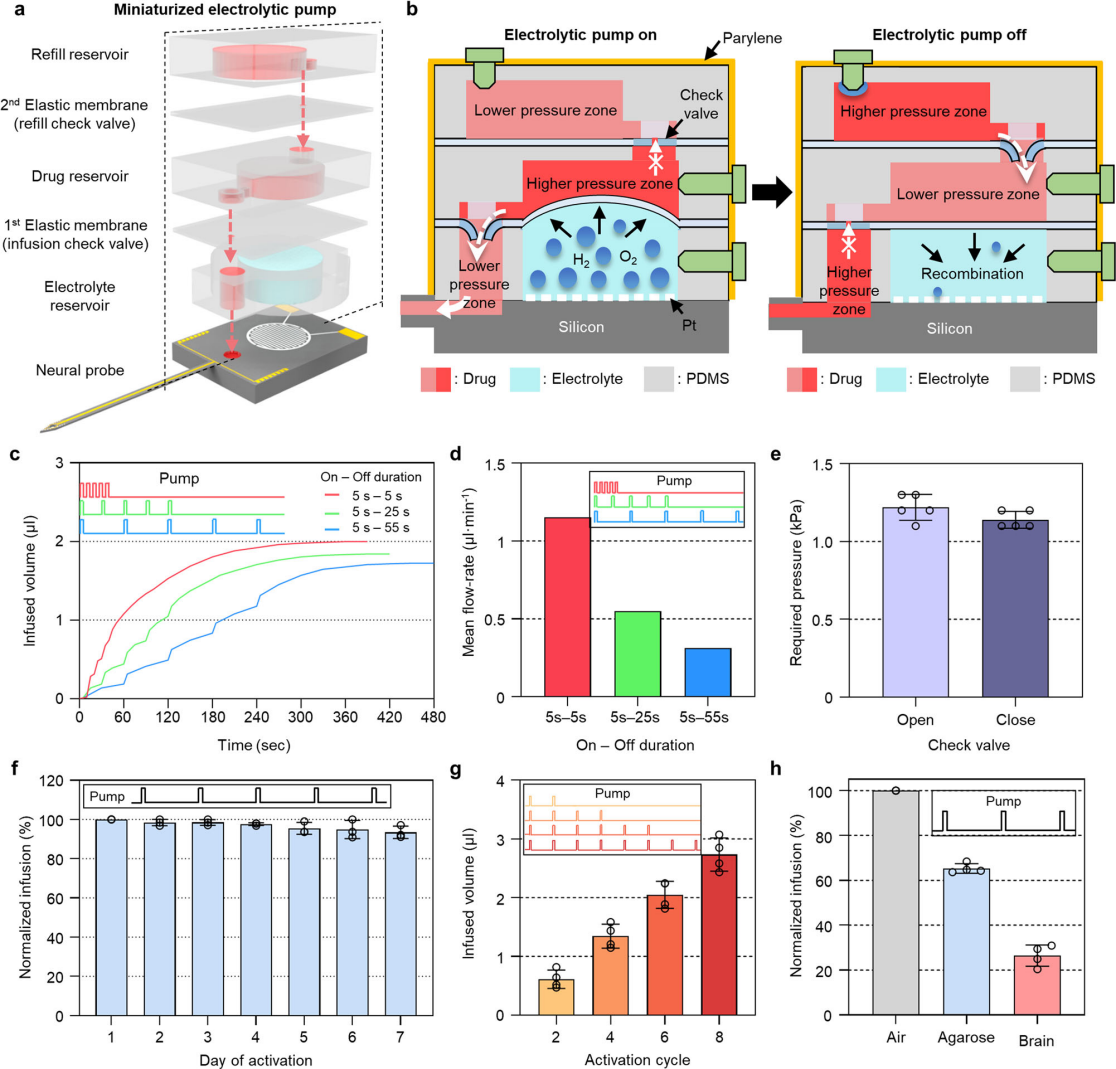
Characterization of the miniaturized electrolytic pump. **a** Exploded view of a miniaturized electrolytic pump, consisting of three reservoir layers, two elastic membranes with inherently formed check valves, and a drug deliverable neural probe with the interdigitated electrode (IDE). **b** Series of schematic illustrations showing sequential on-off operations of the electrolytic pump. **c** Infused volume over time by activating 5 cycles with 10 s (5 s on and 5 s off), 30 s (5 s on and 25 s off), and 60 s (5 s on and 55 s off) period. The sketch inset in the graph shows the on and off times of the pump over a whole period. **d** Mean flow rate according to activation periods of 10 s (5 s on and 5 s off), 30 s (5 s on and 25 s off), and 60 s (5 s on and 55 s off). **e** Pressure required to open and close the check valves formed in the electrolytic pump (*n* = 5). **f** Normalized infusion during the 7 days of pump activation (5 cycles; 5 s on and 55 s off over a 60 s period) (*n* = 3). **g** Infused volumes according to the activation cycles of 2, 4, 6, and 8 cycles (5 s on and 55 s off over a 60 s period) (*n* = 4). **h** Normalized infusion according to the infusion media of air, agarose, and brain (3 cycles; 5 s on and 55 s off over a 60 s period) (*n* = 4). The sketch inset in the graphs shows the on and off times of the pump over a whole period. Data are presented as mean values ± SD with individual data points. *n* is the number of samples.

The electrolytic pump on the neural probe consists of three reservoir layers, two elastic membranes, and IDE on the neural probe. The reservoir layers and the elastic membranes were made of PDMS. The three reservoir layers included an electrolyte reservoir at the bottom, a drug reservoir in the middle, and a refill reservoir at the top. The two elastic membranes were placed between the layers, and the membranes separated the reservoirs and also formed check valves that allowed only unidirectional flow through the opening, which had a diameter of ~0.2 mm. The check valves in the middle of the fluidic channels are required for the electrolytic pumps to provide reliable delivery of the drug by preventing drug infusion and backflow during the resting states of the pump. Especially for in vivo applications, the absence of check valves may induce tissue suction by the backflow, causing channel clogging that results in degradation of the performance or even the failure of the device^24,25^. To ensure reliable drug infusion during the repeated activations of the pump, we had to eliminate the bubbles that adhered to the IDE after every operation of the pump. If the bubbles on the IDE are not removed properly, the effective area of the IDE exposed to the electrolyte would decrease, which would prevent the membrane from going to the initial position for the next injection of the drug. In addition, the pressure would build up within the enclosed electrolyte reservoir as the pump operates, and this may damage the membrane of the pump. Thus, we coated a polymer layer, i.e., Nafion (thickness = 3683 ± 257 Å), on the IDE (Supplementary Fig. 6). The recombination of gases is catalyzed by Pt IDE, and it can be accelerated by the Nafion layer, which facilitates the diffusion of gases due to its high gas solubility^26^. In addition to the bubbles on the IDE, the bubbles generated in the drug reservoir may cause a loss of pressure for successive drug injections. In the case of a mechanical pump, such as iPrecio which was utilized by Dagdeviren et al., it is operated by a micro-motor and may provide the accurate and reliable injection of drugs. However, there is a limitation on the miniaturization of the mechanical pump (Supplementary Table 1). Also, the noise produced during its operation may affect behavioral experiments with sensitive animals. We believe that our electrolytic pump could overcome these by miniaturizing the pump enough to be applied to small animals and by suppressing the formation of bubbles in the drug reservoir by both the refill chamber and the check value, which help provide reliable and repeatable delivery of drugs. In addition, the electrolysis-based pump can provide superior performance in power consumption and preservation of drugs compared to the heat-based pump (Supplementary Table 1). The three reservoir layers and the two elastic membranes were stacked on the body of the probe while aligning the IDE within the electrolyte reservoir and securing the flow path to the inlet (Fig. 2a). With this configuration, the miniaturized electrolytic pump operates as follows (Fig. 2b). When the pump is activated, electrolysis occurs, forming oxygen and hydrogen gases that generate pneumatic pressure and push the membrane. Then, the infusion check valve between the bottom and top layers is opened, which induces drug infusion while the refill check valve between the middle and top layers is closed, thereby blocking the fluidic channel between the drug reservoir and the refill reservoir. When the pump is deactivated, the generated gases quickly recombine into the water due to the Nafion, allowing the deflected membrane to recover, which induces backpressure to the drug reservoir from the outlet of the probe. However, the infusion check valve is closed to prevent any backflow. Also, the refill check valve is opened, which allows the drug reservoir to be refilled from the refill reservoir through the hydrophilic channel, thereby eliminating the pressure difference between the drug reservoir and the refill reservoir. Air can flow into the refill reservoir through the microscopic gaps around the plugged inlet port. Therefore, the negative pressure induced by the gas recombination can be compensated. The bubble is formed in the refill reservoir, but it cannot block the check valve because the bubbles are formed mostly from the top of the refill chamber through the gaps around the inlet port. Also, the modification of the surface by the O_2_ plasma treatment during the fabrication process made it hydrophilic, thereby preventing the bubble from blocking the path of the fluid. Throughout the experiments, we activated the pump by applying pulses of 3.3 V for 5 s, which sufficiently infused the filled drug in the reservoir with low power consumption (~6 mW) (Supplementary Fig. 7 and Supplementary Movie 2; the detailed assembly method is provided in the Methods section). As described above, we successfully integrated the reliable and miniaturized pump into the neural probe. Our miniaturized pump has the unique feature of having a refill chamber with a check valve that prevents the formation of bubbles in the drug reservoir. The dimensions of our pump, including the drug volume/limits, were determined considering (1) its integration with the neural probe, (2) the alignment margins during layer-by-layer stacking, and (3) the installation on the head of a mouse. We tried to minimize the weight and size of the pump while ensuring that the pump had enough volume for chronic drug delivery.

### Characterizations of the neural probe integrated with the miniaturized electrolytic pump

To evaluate the performance of the neural probe integrated with the miniaturized electrolytic pump, we characterized each function of both the microelectrodes and the electrolytic pump. First, we measured the electrical impedance of the 16 microelectrodes before and after Pt black electroplating. The average impedance of the Pt electrodes before electroplating was 1.671 ± 0.104 MΩ at 1 kHz, whereas that of the Pt black electrodes after electroplating effectively was lowered to 0.016 ± 0.002 MΩ at 1 kHz. The low impedance was achieved due to the increased effective surface area, which lowers the level of the electrical noise, improves the signal-to-noise ratio, and allows efficient recording of the neural signal^26,27^ (Supplementary Fig. 8).

Next, we characterized the drug infusion capability of the neural probe integrated with the electrolytic pump in vitro. We prepared the fabricated electrolytic pump and the neural probe to measure the infused volume with the hydraulic resistance of fluidic channels. Then, the outlet of the pump and the inlet of the fluidic channels in the neural probe were connected by a tube. While filling the reservoirs and the fluid tube with DI water, an air bubble with a volume of ~1 μl intentionally was placed within the fluid tube so that we could measure the volume of the injected fluid by the displacement of the bubble at various activation conditions. Cyclic activation of the pump was chosen because the infusion with a constant on-time was prone to tissue damage and overdosing, as well as the tearing of the membrane and bursting of the pump due to the drastic increase in the pressure^28^. First, we analyzed the infused volume over time by activating five cycles at various 10-s periods (5 s on and 5 s off), 30 s (5 s on and 25 s off), and 60 s (5 s on and 55 s off) (Fig. 2c). The total infused volumes were measured differently as 2.0, 1.8, and 1.7 μl for the periods of 10 s (5 s on and 5 s off), 30 s (5 s on and 25 s off), and 60 s (5 s on and 55 s off), respectively, even with the same on-time values. Thus, we measured the infusion by residual pressure during the off states for the periods of 10 s (5 s on and 5 s off), 30 s (5 s on and 25 s off), and 60 s (5 s on and 55 s off), and the corresponding infused volumes with settling time were measured as 1.1 μl for ~175 s, 0.7 μl for ~160 s, and 0.4 μl for ~120 s, respectively (Supplementary Fig. 9a). These results showed that the residual pressure might induce differences in the infusion volume, and we can control the infusion volume accurately with consideration of the residual infusion. The mean flow rates were measured as 1.15, 0.55, and 0.31 μl ∙ min^−1^ for the periods of 10 s (5 s on and 5 s off), 30 s (5 s on and 25 s off), and 60 s (5 s on and 55 s off), respectively (Fig. 2d). The flow rates were not higher than those of the previously reported intracerebral drug delivery system, indicating that tissue damage can be minimized irrespective of the activation period^15^.

Also, we measured the flow rates at various on-times (3, 5, 7, and 9 s) while keeping the off-time (55 s) and the number of activation cycles (five cycles) fixed. We found that the DI water in the fluidic tube started to move from the on-time of 5 s and observed that the flow rates increased as the on-times increased (Supplementary Fig. 10a). In addition, based on the hydraulic resistance of the probe and measured flow rates, we estimated the pressure generated at various on-times (Supplementary Fig. 10b and Supplementary Note 1). The estimated pressure was 7.37 kPa at 5 s on, 15.87 kPa at 7 s on, and 18.15 kPa at 9 s on, respectively (Supplementary Fig. 10c). Thus, we confirmed that the on-time of 5 s was the minimum activation time required for initiating the infusion of the drug to the outlet of the probe (Supplementary Movie 2 and Supplementary Fig. 10), and the pressure could be controlled according to the activation time of the pump (Supplementary Fig. 10). Also, we tested the on-time of 10 s, but it was prone to the pump’s bursting and the membrane’s tearing due to the build-up of high pressure. For the in vivo experiment, we chose the on-time of 5 s and activation period of 60 s (5 s on and 55 s off) to minimize cell displacement through slow injection of the drug. Cell displacement means that cells near the electrodes are pushed temporarily by the rapid injection of the drug^6,7^. In particular, if the drug were to be injected rapidly, it could disturb the continuous monitoring of the neural signals from the microelectrodes after the injection of the drug^6,7^. During the operation of the pump, the pump demonstrated prompt initiation of drug infusion and was free of backflow due to the reliable operation of the check valve with its small opening pressure (1.22 ± 0.08 kPa) and small closing pressure (1.14 ± 0.05 kPa) (Fig. 2e).

Also, we characterized the chronic reliability and dose controllability of the pump. To validate the chronic reliability of the electrolytic pump, we measured the infusion volume daily at the same operating conditions (five cycles; 5 s on and 55 s off) for 7 days to mimic the long-term investigation of the effect of the drug. During 7 days of activation with four devices, we observed that the infused volume was maintained within 7% of the initial volume (Fig. 2f). We achieved reliable drug infusion by eliminating the bubbles that were generated in both the electrolyte reservoir and the drug reservoir. Specifically, we tried to eliminate bubbles in the electrolyte reservoir by coating Nafion on Pt IDE, which promotes the recombination of the gas^29^. The accelerated recombination of the gas allows the rapid recovery to the initial state and enables the next electrolysis to generate a constant pneumatic pressure, acting as a reliable pumping source. Next, we integrated check valves and a refill chamber to minimize the formation of bubbles in the drug reservoir. When the pump was deactivated, the infused volume should be compensated in response to the backpressure generated by the gas recombination in the electrolytic reservoir. In the case of the normal electrolytic pump without a refill chamber and the refill check valve, the backpressure was compensated by the formation of an air bubble within the drug reservoir through gas permeable PDMS walls. Thus, the repeated operation of the normal pump resulted in enlarging the air bubble in the drug reservoir, thereby showing the degradation of the infusion performance due to the loss of pneumatic pressure by air bubble compression (Supplementary Fig. 9b). However, the proposed electrolytic pump allowed the drug reservoir to be refilled from the refill reservoir, which released the backpressure. Since backpressure was the main driving force that refilled the drug in the drug reservoir after the injection, the drug reservoir could be refilled irrespective of the amount of drug that remained in the refill reservoir, even in the later activation cycles. In addition, the refill check valve isolated the drug reservoir from the refill reservoir, thereby preventing the air bubbles in the refill reservoir from affecting the infusion characteristics. Next, we analyzed the infused volume according to the number of activation cycles to validate the controllability of the dosage. The electrolytic pump was activated at various cycles, and we found that the infused volume was linearly proportional to the number of activation cycles (*R*^2^ = 0.94), which confirmed that we could accurately control the dosage of the drug in real time (Fig. 2g). During the operation of the pump, backflow was not observed regardless of the different flow rates and infusion volumes due to the integrated check valve. Thus, we demonstrated that the injection volume could be precisely controlled by changing the activation time and the number of activation cycles without backflow.

Finally, we analyzed the infusion characteristics according to the infusion medium to estimate the volume infused into the mouse brain by operating the pump with three cycles (5 s on and 55 s off). Compared with the probe shank in air, the infused volume was decreased to 65 ± 2% in agarose gel (1 wt%) and to 26 ± 5% in the brain of the mouse, respectively (Fig. 2h). We believe that the fluidic resistance, which is determined by the medium surrounding the outlet, affected the flow rate of the pressure-driven electrolytic pump. Furthermore, we monitored the infused volume for 5 days in the brain to monitor the change of the infused volume by channel clogging due to biofouling. The infused volume was kept constant under the same pump operating condition (three cycles; 5 s on and 55 s off) for 5 days (Supplementary Fig. 11). Thus, we concluded that channel clogging might not be significant and achieved reliable drug infusion even for 5 days with implantation of the probe in the brain. Throughout the characterization, we confirmed that the miniaturized electrolytic pump could deliver an accurate amount of drug directly into the brain chronically with low-voltage operation and low consumption of power. Refill can be conducted whenever necessary based on the visual inspection of the reservoirs. The animal should be anesthetized before conducting the refill process. Then, both the cured adhesive (Kwik-Sil, World Precision Instruments (WPI), USA) and the plug in the inlet port of the reservoir are removed carefully. After using a syringe to refill the chamber, we plug the reservoir and cast adhesive again.

### Design and operation principle of the wireless bi-directional communication module

The bi-directional wireless communication module is composed largely of two parts, i.e., (1) a signal recording part for sending neural signals and (2) a pump control part for receiving signals for the operation of the pump (Supplementary Fig. 12a). We used dedicated Bluetooth communication modules for each part to enable the simultaneous operations of the neural signal recording through a PC and the pump control through a smartphone (Supplementary Fig. 12b). Also, the overall system was designed to be light-weighted by integrating electrical components on a thin 0.5 mm printed circuit board (PCB). The signal recording part consists of a signal amplifier chip (RHD2132, Intan Technologies, USA) that amplifies the recorded neural signals from the 16 microelectrode array, an MCU (TI MSP 430, Texas Instruments, USA) for processing recorded signals, and a Bluetooth transceiver (PAN1326B, Panasonic Electronic Components, USA) for wireless transmission of the processed signal data to a Bluetooth receiver connected to the PC (Supplementary Fig. 12b). We minimized the power consumption by adopting low-power electronic components. Also, to overcome the limitation of the number of measurable channels due to the low bandwidth (<1 Mbps) of the Bluetooth communication protocol, we applied a real-time spike detection protocol in the system^30^. By only transmitting neural spikes above the noise level (~50 μV) with timing information by the MCU, we were able to measure neural signals from 16 electrodes simultaneously.

The pump control part consists of a BLE module embedded with MCU (FBL770BC, Firmtech, South Korea) and a p-channel MOSFET for a stable power supply during the operation of the pump. We can control the pump using an Android-based smartphone through the application provided by the manufacturer (Supplementary Fig. 12b). Thus, we were able to initiate or stop infusion and wirelessly control the dose of the drug that was delivered at any time. The power consumption of the bi-directional communication module was 74 mW during the transmission of the recorded neural signal and 5 mW for the wireless pump control (Supplementary Table 2).

The weight and size of the bi-directional wireless module were 2.36 g and 1350 mm^3^, respectively, which was sufficiently small to be applied to the head of a mouse^31^ (Supplementary Table 2). In addition, due to the low consumption of power, the system could operate for about an hour using a 30 mAh lithium polymer (Li-Po) battery that weighed only 0.94 g. The weight of the proposed system is slightly higher than wireless systems that only have stimulation capability, but the weight is similar to that of the previous wireless recording system even though the proposed system has an additional wireless pump module (Supplementary Table 2). Also, our system available could be applied to social experiments that require the simultaneous recording of the neural signal among multiple mice and the delivery of the drug using the bi-directional wireless module based on the BLE protocol that minimizes signal interference among wireless systems.

### Wireless drug delivery and simultaneous monitoring of neural and behavioral changes

**Fig. 3:**
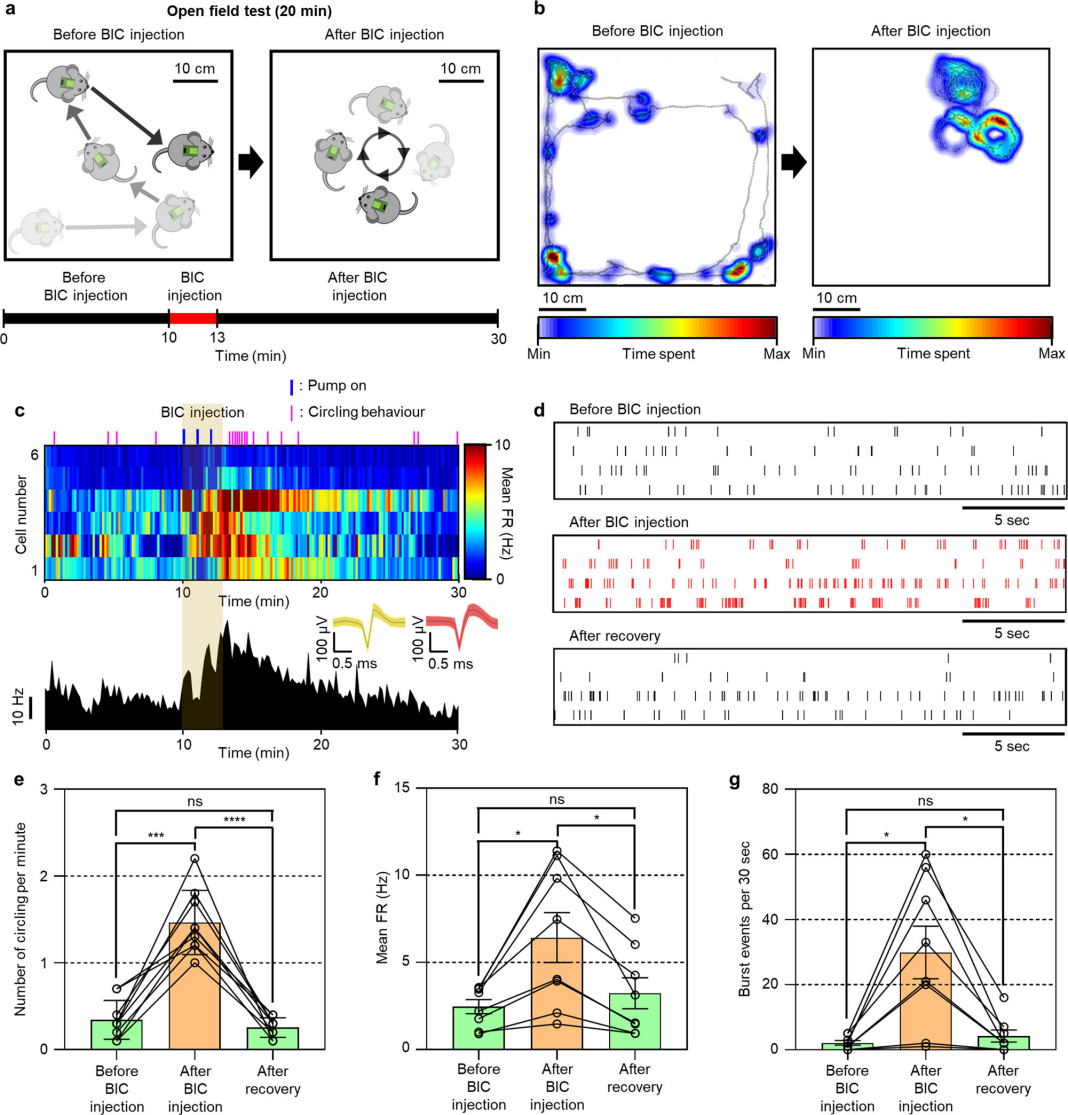
Wireless drug delivery and simultaneous monitoring of neural, behavioral changes. **a** Series of schematic illustrations showing the behavioral change induced by the injection of bicuculline (BIC) into the substantia nigra (SN) and experimental timeline. **b** Series of heatmaps showing the behaviors of a mouse before and after the BIC injection (5 min period). **c** Real-time circling behavior and neural activities of SN neurons and merged firing rate of SN neurons in accordance with BIC injection. The blue line above the heatmap indicates the time at the pump on (3 cycles; 5 s on and 55 s off over a 60 s period). The pink line above the heatmap indicates the time at which the circling behavior appeared. The inset shows representative neural signals. **d** Neural firing patterns of SN neurons before and after BIC injection as well as after recovery. “After recovery” was based on the time when the compulsive circling behavior changed to normal behavior (e.g., walking behavior). **e** The number of circling per minute before and after BIC injection, and after recovery (*n* = 9 where *n* is the number of repetitive experiments with the 3 mice (i.e., three experiments per mouse); *F*(1.193, 9.547) = 63.64, *p* = 0.00001029). **f** Mean firing rates of SN neurons that showed firing pattern change before and after BIC injection as well as after recovery (*n* = 8 where n is the number of neurons from 3 mice; *F*(1.442, 10.09) = 12.31, *p* = 0.00326). **g** The number of burst events for 30 s of some SN neurons before and after BIC injection as well as after recovery (*n* = 8 where n is the number of neurons from 3 mice; *F*(1.074, 7.520) = 13.31, *p* = 0.00670). Data are presented as mean values ± SD with individual data points. Statistical analyses were performed by one-way repeated measures ANOVA with Tukey’s multiple comparisons test. *p* < 0.05 was considered significant. *p < 0.05, ***p < 0.001, ****p < 0.0001. ns no statistical significance.

The key advantage of our system is its ability to perform wireless drug delivery, electrophysiology, and behavior monitoring simultaneously. To demonstrate the simultaneous monitoring of both behavior and neural signals by the drug that was delivered, we implanted the neural probe in the substantia nigra (SN) of C57Bl6 wild-type mice and delivered bicuculline (BIC), a GABA_A_ receptor antagonist, into the targeted region in the brain (detailed surgical procedure are provided in the Method section, Supplementary Fig. 13, and Supplementary Movie 3). It is well known from previous studies that injection of BIC into the SN induces both an increase in neural activities and compulsive circling behavior^32,33^. First, we connected a flexible cable to FFC/FPC connectors of the wireless module (Supplementary Movie 4). The flexible cable was connected easily with a little force, because the other end of the flexible cable was rigidly fixed to the probe’s PCB by epoxy. (The detailed process for packaging the probe is provided in the Methods section.) Then, we connected the battery to the power connector of the wireless module (Supplementary Movie 4). For the real-time observation of the effects of the drug, we performed an OFT without drug infusion for 10 min as a control and activated the electrolytic pump wirelessly for three cycles (3.3 V; 5 s on and 55 s off) and infused ~300 nL of 0.24 mM BIC into SN (Fig. 3a). During the injection of BIC, the animal showed a little behavioral change, such as freezing (Supplementary Movies 5 and 6). The operation of the pump or the liquid infusion might have affected the behavior. However, previous research showed that the aCSF (artificial cerebrospinal fluid) infusion did not affect behavioral change^16^. Also, the electrolyte pump does not produce any sound that can be generated by a piezoelectric pump (Supplementary Table 1). Thus, we inferred that the behavioral change during the injection of BIC might have been due to some other reason, and this will be investigated through further studies. After the injection of BIC, we observed the change in the behavior of the mice from exploratory behavior to compulsive circling (Fig. 3b and Supplementary Movies 5 and 6). Also, Through the real-time monitoring of the neural activities, the firing rates of SN neurons were increased after the injection of BIC (Fig. 3c, d and Supplementary Movies 5 and 6). Specifically, the firing rates started to be increased (more than 150% firing rate compared to that before injection) about 1 min after the pump was activated (Supplementary Fig. 14). As the infused volume increased by three on-off cycles of the pump, the firing rates increased gradually. After the injection of BIC was finished, the increased firing rates gradually returned to the rates before the injection of BIC due to the dilution of the BIC in the brain (Supplementary Fig. 14).

We analyzed the changes in the circling behaviors quantitatively, and we analyzed the firing rates of the neural signals before and after the injection of BIC. We observed an increase by approximately a factor of 4 when we compared the number of circling behaviors before and after the injection of BIC (Fig. 3e). Also, after the BIC injection, the mean firing rate increased by a factor of 2 (Fig. 3f). The increase in the firing rate came from the change of firing patterns changing from spontaneous firing to burst firing, which was recovered afterward (Fig. 3d, f and Supplementary Movie 6)^34^. After the injection of BIC, the burst rate was increased significantly, resulting in an increased firing rate, and, later, the changes returned to the baseline level (Fig. 3g). Their behavior also slowly returned from compulsive circling behavior to normal behavior (Supplementary Movie 6). From the in vivo experiment using wireless BIC infusion, we showed that our wireless neural probe system could deliver a drug while monitoring the changes in behavior and neural activities, including the real-time firing patterns.

### Controllable, chronic drug delivery and consequent changes in neural, behavioral activities

**Fig. 4:**
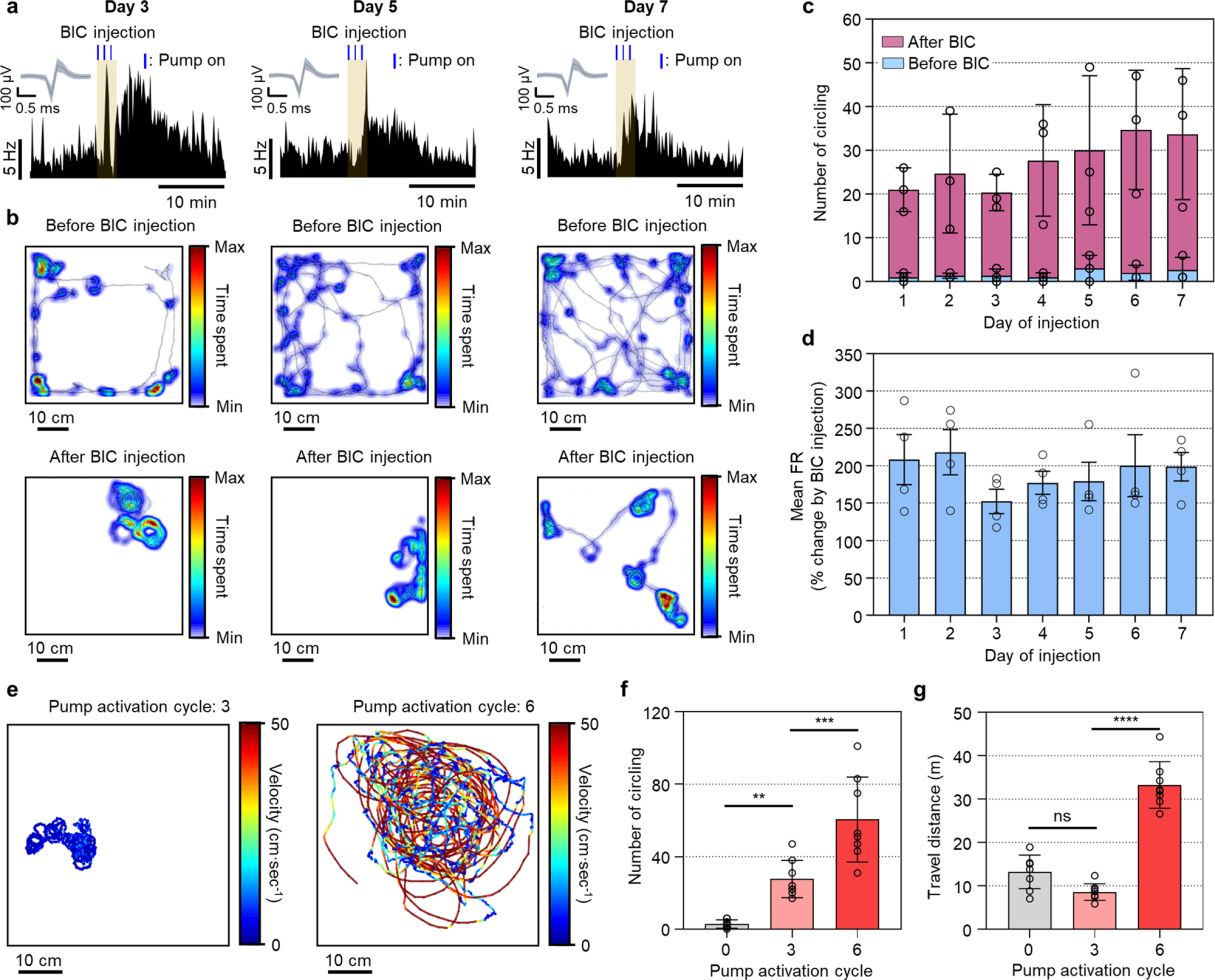
Controllable, chronic drug delivery, and consequent neural and behavioral changes. **a** Firing rates of a representative SN neuron in accordance with BIC injection on day 3, day 5, and day 7. Inset shows a representative neural signal. The blue line above the heatmap indicates the time at the pump on (3 cycles; 5 s on and 55 s off over a 60 s period). **b** Behavior heatmap of a representative mouse in accordance with BIC injection on day 3, day 5, day 7 (5 min period). **c** Number of circling before and after BIC injection daily for 7 days (5 min period; *n* = 3 where *n* is the number of mice). **d** Increase in the mean firing rates after the injection of BIC daily for 7 days (*n* = 4 where *n* is the number of neurons). **e** Representative trajectories with velocity heatmap after BIC injection by activating the pump with 3 cycles and 6 cycles (5 min period). **f** The number of circling after BIC injection by activating the pump with 0 cycle, 3 cycles, and 6 cycles (5 min period; *n* = 8 where *n* is the number of repetitive experiments; *F*(2, 21) = 30.25, *p* = 0.00000065). **g** Travel distance after BIC injection by activating the pump with 0 cycle, 3 cycles, and 6 cycles (5 min period; *n* = 8 where *n* is the number of repetitive experiments; *F*(2, 21) = 87.11, *p* = 0.000000000068). Data are presented as mean values ± SD with individual data points. Statistical analyses were performed by the one-way ANOVA with Tukey’s multiple comparisons test. *p* < 0.05 was considered significant. **p < 0.01, ***p < 0.001, ****p < 0.0001. ns no statistical significance.

Next, we evaluated the chronic reliability and dose controllability of the electrolytic pump of the proposed system. To demonstrate the long-term reliable delivery of the drug, we injected BIC into the SN of a mouse daily for a week using the same pump activation condition described in the previous section. We monitored the neural activity and behavioral changes in real-time according to the daily BIC injection (Fig. 4a, b and Supplementary Movie 7). The number of occurrences of circling behavior as well as the mean firing rate was increased significantly after the BIC injection, and the changes induced by the BIC were consistent for 7 days (Fig. 4c, d).

To demonstrate the dose-controllable drug delivery, we injected a different amount of BIC by activating the electrolytic pump with three and six cycles, respectively. Both conditions induced circling behavior, but the mouse injected with the higher BIC dose (i.e., six-cycle activation) showed more intense circling behavior, rotating with larger circles and higher velocities (Fig. 4e and Supplementary Movie 8). Then, we quantitatively analyzed and compared the behavioral responses to control (no BIC injection), low BIC dose (three-cycle activation), and high BIC dose (six-cycle activation). Consequently, we observed that the number of circling behaviors was proportional to the activation cycle (Fig. 4f). We also observed different travel distances depending on the dosage of the injected BIC (Fig. 4g). Interestingly, the low BIC dose resulted in the least travel distance, and it was even less than the control case. This was due to the relatively slow, somewhat stiffened, circling behavior compared to the freely behaving exploration shown in the control cases. However, the high BIC dose showed a drastic increase in the travel distance due to the intense circling behavior. Through these in vivo experiments, we successfully demonstrated that our system could be utilized in analyzing chronic, dose-dependent drug effects through the real-time monitoring of the changes in the behavioral aspects and the changes in the neural signals of freely behaving mice.

### Modulation of feeding behavior by drug delivery and simultaneous monitoring of changes in the neural signals

**Fig. 5:**
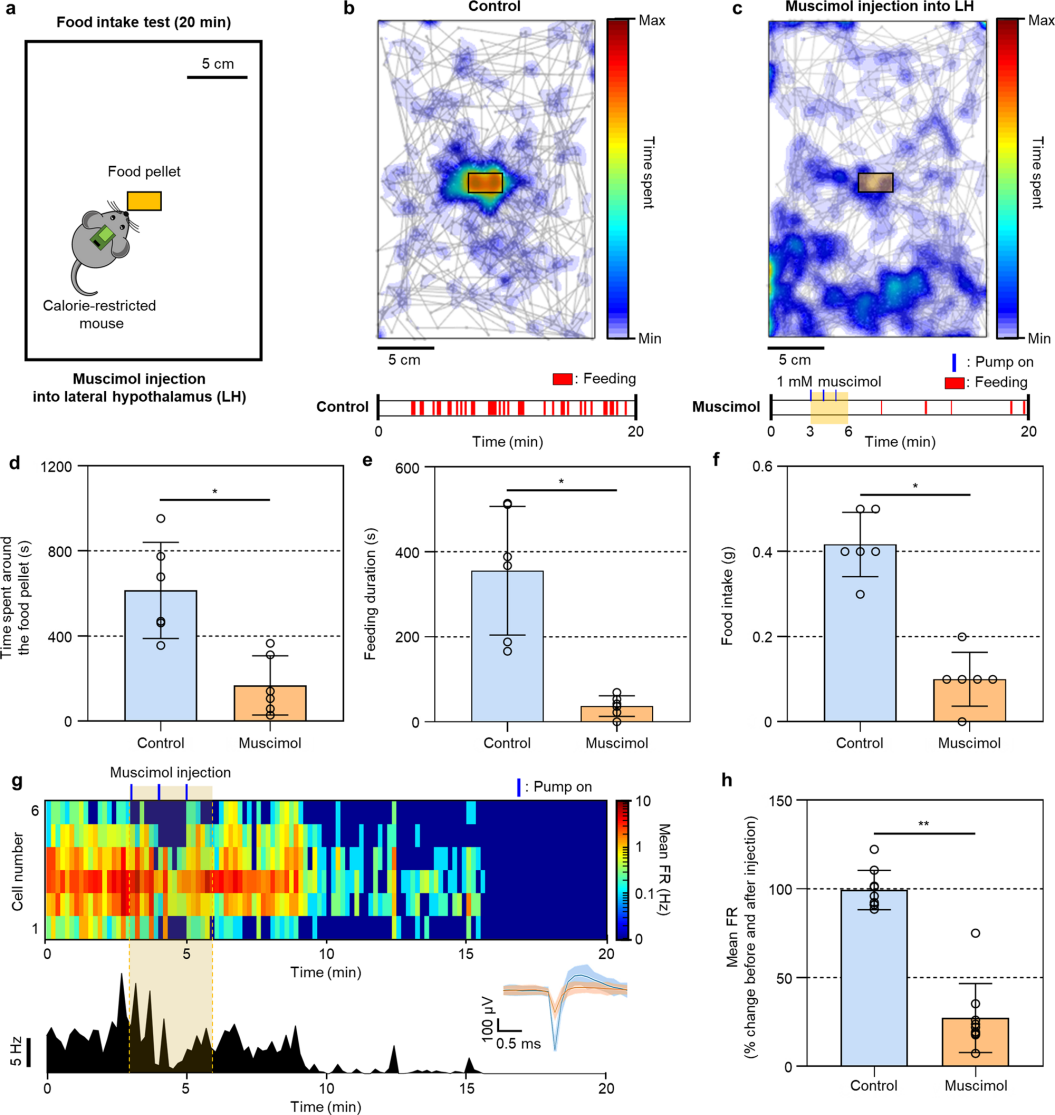
Modulation of feeding by drug delivery and simultaneous monitoring of neural changes. **a** Schematic illustration showing the experimental setup for food intake test. **b**, **c** Behavior heatmap expressed based on nose positions in **b** a control experiment and **c** muscimol-injected experiment. The blue line above the heatmap indicates the time at the pump on (3 cycles; 5 s on and 55 s off over a 60 s period). **d** Comparison of time spent around the food pellet between the control and muscimol-injected experiments (*n* = 6 where *n* is the number of mice; *p* = 0.03125). **e** Comparison of feeding duration between control and muscimol-injected experiments (*n* = 6 where *n* is the number of mice; *p* = 0.03125). **f** Comparison of food intake between the control and muscimol-injected experiments (*n* = 6 where *n* is the number of mice; *p* = 0.03125). **g** Real-time neural activities of LH neurons and merged firing rate of LH neurons in accordance with the injection of 1 mM of muscimol. The blue line above the heatmap indicates the time at the pump on (3 cycles; 5 s on and 55 s off). The inset shows representative neural signals. **h** Change in the mean firing rate of LH neurons depending on the injection of muscimol (*n* = 9 where *n* is the number of neurons; *p* = 0.00391). Data are presented as mean values ± SD with individual data points. All statistical analyses were performed by the Wilcoxon matched-pairs signed rank two-tailed test. *p* < 0.05 was considered significant. *p < 0.05, **p < 0.01.

The wireless drug delivery capability of our system also allows us to modulate the behavior of an animal in natural status, which also can be correlated with the neural response. This feature potentiates the neuropharmacological study of physiological behaviors that are involved in complex neural circuits and require multi-lateral validation. In this experiment, we applied the system to modulate the feeding behavior of freely behaving mice by delivering the drug to the region of the brain related to feeding and correlate with the change in their neural activities. Previous studies have reported that GABAergic neurons in the lateral hypothalamus (LH) manage satiety, and a GABA_A_ receptor agonist, muscimol, modulates the neural circuit related to the feeding behavior^35^. To modulate feeding behavior, we implanted the neural probe system in the LH of wild mice followed by restricted caloric intake for 1 week after recovery. A caloric-restricted mouse was placed in a home cage for 20 min with a food pellet in the center, and the behavior and food intake of the mouse were compared according to the muscimol (1 mM) injection (Fig. 5a, Supplementary Fig. 15, and Supplementary Movie 9). In the case of the mouse without the muscimol injection, feeding behaviors were frequently observed, which resulted in the concentrated nose position around the food pellet (Fig. 5b). However, when muscimol was injected into the LH of the same mouse by activating the electrolytic pump for three cycles (~300 nL delivery), feeding behavior was highly suppressed, and the mouse showed reduced feeding behaviors with the nose position far from the food pellet (Fig. 5c). Throughout the experiments, muscimol injection resulted in the reduced time spent around the food pellet, reduced feeding duration, and reduced consumption of food pellets, clearly indicating the suppressed intake of food (Fig. 5d–f). We also performed real-time monitoring of the LH neurons after the injection of muscimol (Fig. 5g). While the mean firing rate of the LH neurons did not show a significant change before the muscimol injection, we observed an approximately three-fold decrease in the mean firing rate after the muscimol injection (Fig. 5h). This experiment clearly indicated that our system could be applied to the drug-induced neuromodulation with monitoring of the corresponding behavior and electrophysiological change.

### Measurement of neural activity of two mice in competition for food

The significant advantage of a wireless system is the ability to monitor the behaviors of multiple animals without any physical restrictions, which allows the precise observation of social interactions. With wireless drug delivery, we can even modulate socially interacting behaviors between mice. We designed an experiment that effectively showed the modulation of social behavior by drug delivery to the brain. During the competition for food, two mice endeavor to have the same food. However, as shown in the previous section, it is possible to modulate feeding behavior by delivering muscimol to LH. Therefore, we expected that the modulation of feeding behavior on one mouse by drug injection could also affect the social interaction (i.e., from competition to non-competition for food) between two mice during food competition.

During the competition for food prior to the neuromodulation experiment, we measured the neural activities of a pair of freely behaving mice during their competition for food. Previous studies have reported that the medial prefrontal cortex (mPFC) neurons are related to general social behaviors, including recognition^36,37^, interaction^38,39^, and particularly competition^19,40^. Thus, we implanted a neural probe in the mPFC region of a mouse with a green LED attached (i.e., green mouse) to record the neural signal related to social behavior. Next, we implanted a neural probe with drug delivery in the LH region of the mouse with a red LED attached (i.e., red mouse) to both record the neural signal and modulate the feeding behavior. Before the competition for food, both mice experienced caloric restriction to induce proper food competition between them. Also, the mice were trained individually for a week to adapt to the food provision protocol. (The detailed preparation procedures for the competition for food are provided in the Methods section.)

**Fig. 6:**
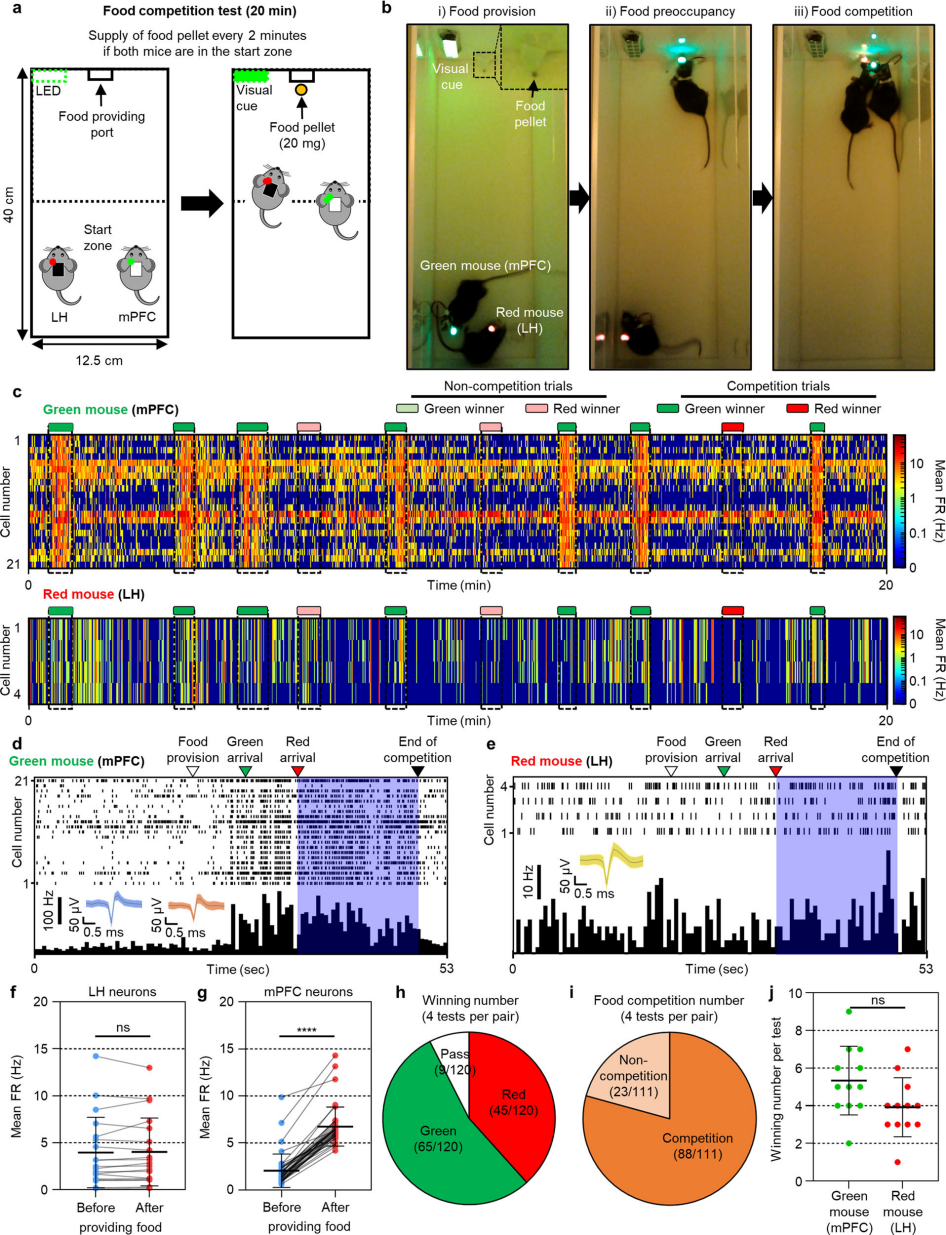
Food competition between two freely behaving mice and real-time monitoring of the neural activities. **a** Schematic illustrations showing the experimental setup and a brief protocol for the food competition test. **b** Sequential photographs captured during a food competition test, showing (i) food provision, (ii) food preoccupancy by one mouse, and (iii) food competition. **c** Neural activities of a green mouse (mPFC neurons) and a red mouse (LH neurons) in accordance with competition and non-competition trials during a representative food competition test. **d** Real-time firing rate of mPFC neurons and **e** LH neurons in accordance with behavioral events during a representative food competition trial. Inset shows representative neural signals. **f** Mean firing rate of LH neurons (*n* = 19 where *n* is the number of LH neurons; *p* = 0.83996) and **g** mPFC neurons before and after proving the food pellet (*n* = 40 where *n* is the number of mPFC neurons; *t*(78) = 10.89, *p* < 0.000000000000001). **h** Overall winning history of three mouse pairs during four food competition tests (120 trials). **i** Overall competition occurrence within the non-passed trials (111 trials). **j** Number of winning per test for each mouse (*n* = 12 where *n* is the total number of games with 3 pairs of mice; *t*(11) = 1.493, *p* = 0.16367). Data are presented as mean values ± SD with individual data points. Statistical analyses were performed by the two-tailed Mann–Whitney test in **f**, the two-tailed unpaired *t*-test in **g**, and the two-tailed paired *t*-test in **j**. *p* < 0.05 was considered significant. ****p < 0.0001. ns no statistical significance.

The food competition was performed in a custom-made activity chamber that measured 40 × 12.5 × 20 cm^3^ and consisted of a port for providing food and a green LED as a visual cue for noticing the provision of food (Fig. 6a). Half of the chamber away from the port where the food was provided was set as a start zone. When both mice were positioned in the start zone, a bite-sized food pellet (20 mg) was provided along with the visual cue, and then the competition began (Fig. 6b and Supplementary Fig. 16). The food pellet was provided only once in a trial, and food was provided only when both mice stayed in the start zone during the entire trial period, which lasted 2 min. (The detailed method and procedure in the food competition test are provided in the Methods section.)

First, we analyzed the real-time neural activities of each mouse during a food competition test (Fig. 6c and Supplementary Movie 10). During a 20-min competition test, the green mouse showed an increase in the firing rates of mPFC neurons after providing the food pellet, but no significant change in the firing rates of LH neurons was observed from the red mouse during the same competition test. In the case of the green mouse, the mPFC neurons were activated continuously, from the moment the mouse saw the food pellet until the end of the competition (Fig. 6d). Specifically, the firing rate of the neural spike started to increase as soon as the green mouse noticed the visual cue and started to run for the food. Also, increased firing rates of the mPFC neurons were maintained when the opponent approached and guarded the opponent’s food pellets. Based on these results, we could infer that the mPFC neurons are involved extensively in social^36–39^ and non-social behaviors^19,41^, such as approach, competition, recognition, and seeking rewards. In the case of the red mouse, the LH neurons did not show any significant change during the same competition trial, indicating that the LH region was not related to competitive behaviors (Fig. 6e). These results were consistent throughout the experiments, and they were confirmed statistically (Fig. 6f, g and Supplementary Fig. 17a).

Then, we statistically analyzed the results of the food competition. Three pairs of mice underwent 120 competition trials (3 mouse pairs × 4 competition tests × 10 trials per test). Throughout the competitions tests, the red mice showed a 37.5% (45/120) winning rate, while the green mice had a winning rate of 54.2% (65/120) winning rate, and the remaining 7.5% (9/120) were classified as passed trials (Fig. 6h and Supplementary Fig. 17b). Within the non-passed trials, the competition occurred at a rate of 79.3% (88/111), while a 20.7% (23/111) rate was observed for the one-sided acquisition of food without competition (Fig. 6i and Supplementary Fig. 17b). In summary, the green mice won more times, but there was no quantitative difference between the number of times each mouse won the competition, which indicated that it was not a one-sided winning (Fig. 6j and Supplementary Fig. 18). Overall, we demonstrated that the food competition protocol did not show one-sided winning, and we were able to measure the neural signals successfully during the competition and to verify the correlation of LH neurons and mPFC neurons with the food competition.

### Modulation of the food competition between two mice by the delivery of the drug

**Fig. 7:**
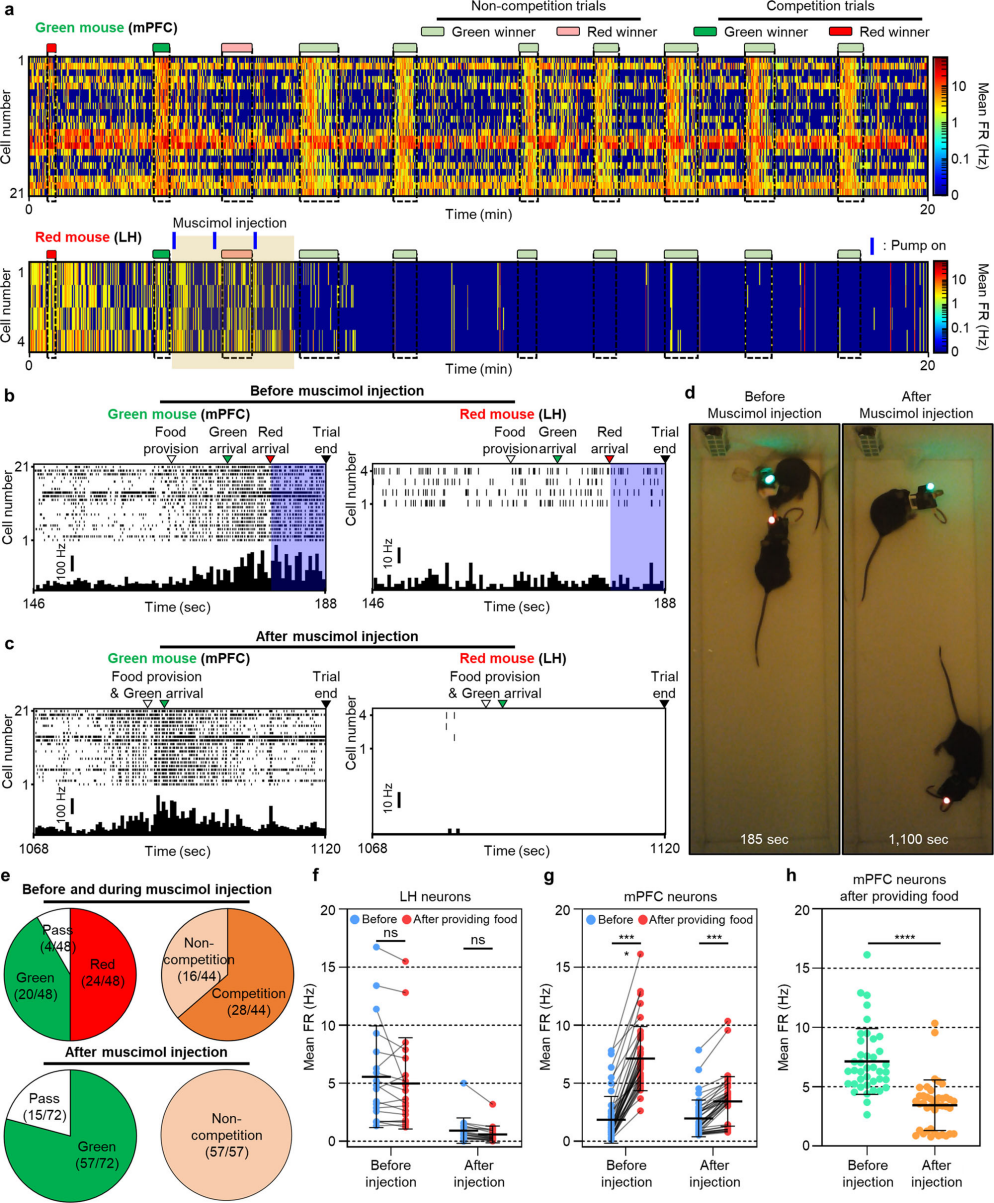
Drug-induced modulation of the food competition and simultaneous monitoring of the neural and social interaction changes. **a** Neural activities of a green mouse (mPFC neurons) and a red mouse (LH neurons) in accordance with competition patterns during a representative food competition test with muscimol injection into the LH of the red mouse. The blue line above the heatmap indicates the time at the pump on (3 cycles; 5 s on and 55 s off over a 60 s period). **b**, **c** Real-time firing rate of mPFC neurons and LH neurons in accordance with behavioral events during a representative food competition trial **b** before and **c** after the muscimol injection. **d** Representative photographs taken before and after the muscimol injection, showing the absence of competition after the muscimol injection. **e** Overall winning history and competition occurrence before, during, and after the muscimol injection during four food competition tests conducted with three pairs of mice. **f** Mean firing rate of LH neurons (*n* = 19 where *n* is the number of LH neurons; before: *p* = 0.72911; after: *p* = 0.13197) and **g** mPFC neurons before and after providing the food pellet depending on the muscimol injection (*n* = 40 where *n* is the number of mPFC neurons; before: *t*(78) = 9.762, *p* = 0.000000003; after: *t*(78) = 3.485, *p* = 0.00081). **h** Mean firing rates of mPFC neurons during the competition depending on the muscimol injection (*n* = 40 where *n* is the number of mPFC neurons; *t*(78) = 6.691, *p* = 0.000000003). Data are presented as mean values ± SD with individual data points. Statistical analyses were performed by the two-tailed Mann–Whitney test in **f** and the two-tailed unpaired *t*-test in **g** and **h**. *p* < 0.05 was considered significant. ***p < 0.001, ****p < 0.0001. ns no statistical significance.

After we confirmed the food competition protocol, we demonstrated the modulation of feeding behavior by the wireless drug delivery to one mouse during the competition test to further highlight the neuropharmacological utility of our system for studying social interactions. From the analysis of the neural signals and the behavioral responses to the in vivo drug delivery, we obtained interesting results, i.e., results that were not feasible by the existing neural interfaces. To modulate feeding behavior in the food competition, we injected muscimol (1 mM; ~300 nL injection by three-cycle activation) into the LH of the red mouse and monitored the changes in neural activities as well as the social interaction (i.e., competition) (Supplementary Fig. 19). During the competition test with the muscimol injection, the mPFC neurons of the green mouse showed an increased mean firing rate during the competition compared to the rate before the competition, as shown in the results in the previous section (Fig. 7a). However, the mean firing rate of the LH neurons of the red mouse decreased after the injection of muscimol (Fig. 7a).

However, after the injection of muscimol into the LH of the red mouse, we found interesting results, i.e., the firing rate of the mPFC neurons increased only after the food pellet was provided (Fig. 7c), while the mPFC neurons showed increased activity during the entire period of the competition before the injection of muscimol (Fig. 7b). The change in the mPFC neurons by the injection of muscimol into the opponent mouse can be explained by the change in social interaction. Before the injection of muscimol, we were able to observe the social interaction between them, such as the approach to the opponent mouse and the competition for the food. However, after the injection of muscimol into the LH of the red mouse, its appetite was suppressed, and their social interaction did not occur (Fig. 7d, Supplementary Fig. 20, and Supplementary Movie 11), which might induce the decrease in mPFC activity after muscimol injection.

Next, we statistically analyzed the winning rates of the two mice according to the muscimol injection (Fig. 7e and Supplementary Figs. 20 and 21). Within the 120 competition trials (3 mouse pairs × 4 competition tests × 10 trials per test), 48 trials were performed before or during the injection of the muscimol, and 72 trials were performed after the injection of the muscimol. Before and during the injection of muscimol, two mice showed similar winning rates, i.e., 50% (24/48) for the red mouse and 41.7% (20/48) for the green mouse. However, after the injection of muscimol, the green mouse won all of the competition except for the passed trials. Moreover, 100% of the non-pass trials were non-competition trials, which indicated that the green mouse acquired all of the provided food without any competition after the injection of muscimol into the red mouse (Supplementary Figs. 20 and 21). These results strongly indicated that the injection of muscimol into the LH successfully suppressed the appetite of the red mouse.

In addition, we statistically analyzed the changes in the neural activity in LH and mPFC according to the muscimol injection. As expected, the mean firing rate of LH neurons was decreased, and that of the mPFC neurons was still increased after the muscimol injection (Fig. 7f, g and Supplementary Fig. 22a–f). However, we found a significant decrease in the mean firing rate of the mPFC neurons (Fig. 7h, Supplementary Figs. 22g–I and 23–25) because there was no social interaction, including competition for the food pellet, after the injection of muscimol. From the food competition experiments, we successfully modulated feeding behavior, i.e., by administering the drug to one mouse, we induced a change in the social interaction, including competition for the food, and we observed changes in the neural activities of both mice, neither of which has ever been demonstrated before.

### Change in the neural activities of mPFC by successive non-participation of the competitor during food competition test

**Fig. 8:**
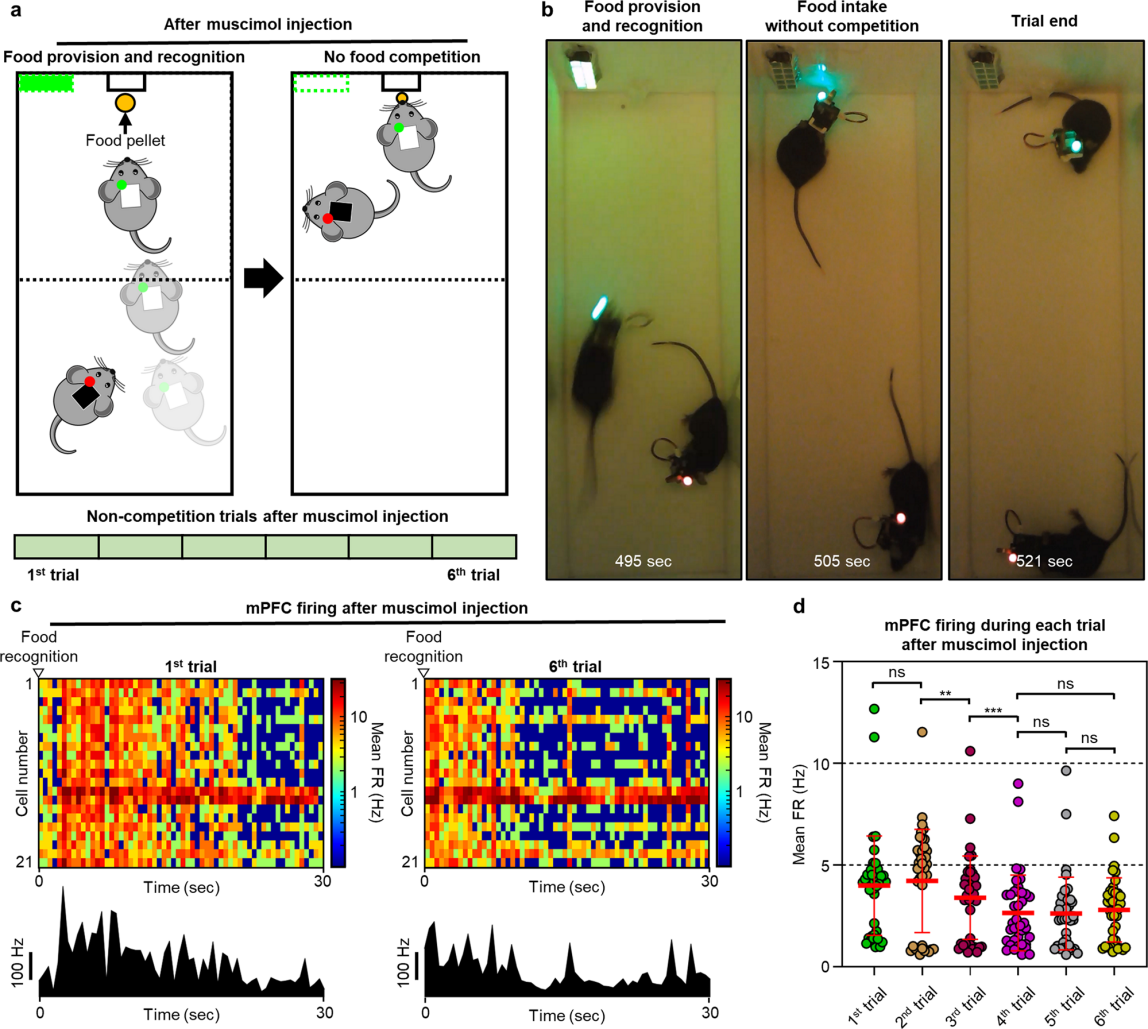
Change in the neural activities of mPFC by successive non-participation in competition of the competitor during food competition test. **a** Schematic illustrations showing the unilateral food winning by the green mouse without the food competition in the successive 6 trials after muscimol injection into the LH of the red mouse. **b** Sequential photographs captured during non-food competition trial after muscimol injection into the LH of the red mouse. **c** Dynamic changes of mPFC neural activities in the green mouse during the first and sixth trials after the injection of muscimol into the LH of the red mouse. **d** Comparison of mPFC neural activities between trials after the injection of muscimol into the LH of the red mouse (*n* = 40 where *n* is the number of mPFC neurons; *F*(2.579, 100.6) = 29.89, *p* = 0.000000000001029). Data are presented as mean values ± SD with individual data points. Statistical analysis was performed by the one-way repeated measures ANOVA with Tukey’s multiple comparisons test. *p* < 0.05 was considered significant. **p < 0.01, ***p < 0.001. ns no statistical significance.

When we further analyzed the firing rate of the mPFC neuron in the green mouse after injecting muscimol into the LH of the red mouse, we found an interesting result, i.e., a change in neural activity. After the injection of muscimol into the LH of the red mouse, the mouse no longer participated in the competition for food, and the green mouse took a food pellet without competition in 6 successive trials (Figs. 7e and 8a, b). Interestingly, the neural activities of mPFC decreased gradually as the number of trials increased despite the fact that the green mouse was experiencing the same non-competitive trials (Supplementary Fig. 26 and Supplementary Movie 12). In all six successive trials, the neural activity of mPFC neurons increased when the green mouse recognized the food pellet and ran to eat it, as with the above result and previous studies^19,41^. However, the mPFC neurons during the first trial after the injection of muscimol showed longer and higher neural activity than those during the sixth trial (Fig. 8c). We also analyzed the mean firing rates of the mPFC neurons for the six successive non-competition trials to investigate changes in the mPFC neurons as the trials proceeded (Fig. 8d and Supplementary Fig. 27). The mean firing rate initially was high for the first two trials, but after the muscimol injection, the mean firing rate decreased gradually until the fourth trial, and it remained low afterward. The gradual decrease of the mean firing rate in the same non-competition trials may indicate that (1) as sessions without competition continued, the green mouse no longer regarded these sessions as competition, (2) the effort for learning the competition rule related to green LED was changed over time, or (3) social interaction was decreased. In addition, we analyzed the time for the green mouse to reach the food pellet during successive, non-competition trials. The average time to reach the food pellet increased slightly over the trials, but there was no statistical difference between the trials (Supplementary Fig. 28). This result could be inferred as the change of motivation for the food.

This in-depth analysis that correlates the activities of the mPFC neurons with social interest against the competitor or the changes of perception against the test or the decrease in social interaction was possible due to the ability to simultaneously observe the neural activities and behaviors in real time while modulating the food competition by injecting the drug into the competitor. Throughout the food-competition experiments, we demonstrated that, by using our system, even the experiments for studying the correlation between the brain and social behaviors caused by the drugs could be performed sufficiently with a high degree of freedom.

## Discussion

Wireless and chronic delivery of a drug with simultaneous recording of neural activities is essential in studies that are designed to evaluate the neuropharmacological effects in free-moving and even socially interacting animals. The neural probe system presented here uses a well-developed electrolytic pump in a miniaturized form factor to provide chronically reliable delivery of controllable doses of the drug. The neural activities were recorded from the microelectrode array integrated on the MEMS neural probe that was designed to reach a region deep in the brain and integrated with microfluidic channels to deliver the drug to the target region. Both the delivery of the drug and electrophysiology were performed wirelessly through a customized wireless module that enabled concurrent, bi-directional communication (i.e., drug-induced neuromodulation and monitoring of the effect at the same time) as well as the stable and accurate selection of target animals among multiple animals that were free to move within the enclosure. Even with the multiple functionalities achieved in a single system, the wireless neural probe system was developed to be miniaturized and to have a light weight, thereby enabling its application to small animals (e.g., mice) with negligible effect on their behaviors. When these features are utilized fully, even neuropharmacological studies on social interactions can be conducted precisely, enabling the identification of neural and behavioral correlations.

In vivo experiments with chronic drug delivery with electrophysiology illustrate powerful application opportunities for the proposed system in behavioral neuroscience research in which neuropharmacological studies are required. We conducted various in vivo experiments to demonstrate the features of our system. The first in vivo experiment demonstrated the real-time monitoring of neural activities during compulsive circling behaviors that were induced by the injection of BIC into the SN of freely behaving mice. Then, we demonstrated the capability of adjusting the dosage of the drug by showing the change of circling behaviors according to various BIC pumping conditions. Also, we showed that the pump has long-term reliability, which means that the pump could induce reliable drug-induced changes in both neural activities and behaviors for 7 days. We also demonstrated the modulation of neural activity by muscimol injection in LH and consequent feeding suppression of caloric-restricted mice. Finally, we demonstrated the modulation of competition between two mice by the suppression of feeding in one mouse through muscimol injection while simultaneously monitoring the changes in neural activities in both mice. We were able to leverage the new wireless drug delivery system with electrophysiology to observe the change in neural activity of mPFC of a mouse to the behavioral change of the other mouse that was induced by the muscimol injection. It might be inferred that the participating mouse that was not modulated by the drug no longer regarded these sessions as competition since the sessions continued without competition. Although this result must be confirmed through further in vivo experiments, it is evident that the proposed wireless neural probe system enabled new behavioral experiments that were not feasible with current neural probe systems.

The proposed system enables (1) the investigation of how neurons respond depending on the various doses of a drug or chronic exposure to a drug and (2) the determination of the behavioral changes that are induced in freely behaving animals. In addition, researchers can monitor the drug efficacy precisely in a group of animals by correlating the neural activities of a certain brain region with various behavioral patterns, including social interactions. Applications of our system will be extended further by the addition of additional functionalities. For instance, the integration of a flow meter will allow the measurement of the exact volume of the drug that is delivered^42^. Also, the integrated flow meter will make it possible to check the channel clogging caused by a chronic immune response in a long-term experiment. Also, an additional drug reservoir will enable us to induce variable behavioral changes using multiple drugs^15^ or to control the concentrations of drugs by injecting them simultaneously with a buffer solution^6^. It is noteworthy that our system demonstrated unprecedented applications, suggesting an advanced experimental paradigm in behavioral neuropharmacology. In addition, we expect that the low bending stiffness of the probe with small dimensions will show long-term recording stability similar to that of the previous probe^43^. Also, the surface coating technique will improve the short-term and long-term recording quality of the probe^44^ and allow the monitoring of the signal changes by the drug from many neurons over a long period of time. Furthermore, the use of short and thin cables for the connections to the system will allow us to minimize the physical disturbance of other mice by the cable and reduce the weight of the system. We expect that our system will be utilized as an effective tool for the screening of neurological drugs and that it will contribute to the development of pharmacotherapy for various neurological disorders.

## Methods

### Fabrication of the neural probe embedded with microfluidic channels

The neural probe was fabricated through the previously developed processes^7^. Briefly, the microfluidic channels within a silicon-on-insulator (SOI) wafer (4-inch SOI wafer, Buysemi, South Korea) were formed by deep reactive ion etching (DRIE), anodic bonding of a glass wafer, chemical-mechanical polishing, and thermal reflow processes. Electrodes for the recording of neural activity and IDE for the electrolysis were formed by successive processes of metal deposition and etching. Finally, the neural probe was released from the SOI wafer by consecutive DRIE and RIE processes.

### Fabrication of the miniaturized electrolytic pump

The miniaturized electrolytic pump consists of three reservoir layers and two elastic membranes, all of which were fabricated using PDMS (Sylgard 184, Dowhitech Silicone Co., South Korea). For the bottom layer, a 2-mm-thick acrylic sheet was laser machined to fabricate a mold then degassed PDMS (10:1 mixture) was cast, and this was followed by clamping with polyester (PET) films (100-micron A4 OHP Film, Jong IE NARA, South Korea) supported by a glass slide. The PET film was used to peel the cured PDMS off easily without any damage. After curing for 30 min in an oven at 80 °C, the parts that comprised the bottom layer were retrieved, and this was followed by the formation of two through-holes for the drug flow path and the electrolyte reservoir that were Ø 1.5 and Ø 5 mm, respectively. The middle and top layers were fabricated through soft lithography. To fabricate the mold for the middle and top layers, SU-8 50 (SU-8 50 Photoresist, MicroChem Corp, USA) was patterned on a Si wafer (4-inch silicon wafer, Buysemi, South Korea) using the photolithography process to form an array of Ø 1.5 mm ring-shaped patterns connected with a Ø 5 mm pattern, and then the 1.3-mm-thick Ø 5 mm acrylic cylinders were attached on every Ø 5 mm pattern. Then, degassed PDMS (10:1 mixture) was cast on the mold to be 1.8-mm thick. The cured PDMS was peeled off and cut into pieces that were 7 mm wide, 9 mm long, and 1.8 mm thick as the middle and the top layer that inherently have a lid. On the lid of the top layer and on the side walls of the middle and the bottom layers, 0.75 mm through-holes were formed for the inlet port of each reservoir. The holes were formed by manually punching the desired spots using biopsy punches (Rapid-Core – 0.35 mm; 0.5 mm; 0.75 mm, WellTech, Taiwan). The parts of the pump were coated with parylene that was 4-μm thick to minimize the evaporation of the drug within the reservoirs^15^. Before fabricating the thin PDMS membrane, a Si wafer was silanized by placing it in a vacuum desiccator with two drops of Trichloro(1H,1H,2H,2H-perfluorooctyl)silane (448931, Sigma-Aldrich, USA) for 30 min. On the prepared silanized Si wafer, degassed PDMS (10:1 mixture) was spin-coated at 500 rpm for 10 s followed by 3500 rpm for 30 s, resulting in a PDMS membrane that was about 20-μm thick, which was used for both elastic membranes. The PDMS membrane was bonded between the middle and the top layers by O_2_ plasma treatment. During the plasma treatment, a Ø1.2 mm magic tape (122A, 3M, USA) was attached temporarily to the center of the ring-shaped pattern to prevent bonding with the membrane. After bonding the PDMS membrane, the center of the ring-shaped pattern was penetrated with a 30 G needle (Shin Chang Medical, South Korea), forming an opening for the check valve (Fig. 2b).

### Assembly of the neural probe integrated with the electrolytic pump

After the neural probe and the parts of the pump were prepared, the neural probe was attached to a custom PCB (fabricated from HANSAEM DIGITEC in South Korea) using an instant adhesive (Loctite 401 liquid super glue, LOCTITE, Germany). To connect the wireless communication module, FFC/FPC connectors (503480-1000, 503480-1200, Molex, USA) were soldered to the front and back sides of the custom PCB. Then, an electrolyte reservoir was bonded on the body of the neural probe using O_2_ plasma while aligning the two through-holes with both the drug inlet and the IDE of the probe body, as shown in Fig. 2b. After plasma bonding, 1% Nafion in isopropyl alcohol (diluted from 5% Nafion; 510211-25ML, Sigma-Aldrich, USA) was drop cast on the IDE, which was dried in an 80 °C oven for 30 min, forming a Nafion film on the IDE. The thickness of coated Nafion was measured at three points per sample from three samples using an alpha step surface profiler (ASIQ, KLA-TENCOR, South Korea). Then, we bonded wires for the electrical connections between the pads of the neural probe and the custom PCB. After connecting two flexible flat cables (fabricated by DAWANFLEX in South Korea) to the FFC/FPC connectors on the custom PCB, the wire-bonded area was protected by thermal epoxy (EPO-TEK 320, Epoxy Technology, Inc., USA). Likewise, the flexible cables connected to the FFC/FPC connectors were fixed on the custom PCB by the thermal epoxy. After curing the thermal epoxy in an oven, the middle and the top layers of the pump parts (i.e., first elastic membrane, drug reservoir, second elastic membrane, and refill reservoir) were bonded on the electrolyte reservoir by O_2_ plasma while manually aligning each part, as shown in Fig. 2b. Finally, using 1 ml syringes with 27 G needles (Shin Chang Medical, South Korea) installed, the electrolyte reservoir was filled with distilled water, and the drug was filled in the remaining reservoirs, after which the ports were sealed with 3D-printed plugs and silicone elastomer (Kwik-Sil, WPI, USA).

### Assembly of the wireless systems with neural probe and wireless module

The overall system consists of a neural probe integrated with an electrolytic pump, a wireless bi-directional communication module, and a custom crown. The neural probe and the wireless module were connected through two flexible flat cables, and they were mounted on the head of a mouse with the custom crown. The crown was designed to fit the neural probe system tightly while encasing the components to protect them. The crown was fabricated by a 3D printer (Ultimaker 2+, Ultimaker, Netherlands) using polylactic acid filament, which is known as a biocompatible polymer.

### Pt black electroplating and impedance measurement

We electroplated Pt black on the Pt microelectrodes to improve the quality of their recording. The Pt black plating solution contained 3% hexachloroplatinic acid hydrate (520896-5G, Sigma-Aldrich, USA), 0.025% HCl (4090-4400, DAEJUNG, South Korea), and 0.025% lead acetate (316512-5G, Sigma-Aldrich, USA) in deionized water^17^. The tip of the neural probe was immersed in the plating solution with a reference electrode (Ag/AgCl wire) and a counter electrode (Pt wire). The Pt microelectrodes were electroplated selectively by applying the electrical potential (–0.2 V, 35 s) through a potentiostat (PalmSens3, PalmSens, Netherlands).

To measure the impedance of the Pt black microelectrodes, we immersed the tip of the neural probe in 0.1 M phosphate-buffered saline solution (21-040-CV, CORNING, USA) with the reference electrode (CHI 151, CH Instruments, Inc., Austin, TX, USA). The impedances of the 16 microelectrodes were measured by a frequency sweep mode (10 Hz–10 kHz) using an impedance analyzer (nanoZ, Neuralynx, Bozeman, Montana, USA).

### Animal preparation

All of the procedures, including the use of animals, were approved by the Korea Institute of Science and Technology (KIST) in Seoul Korea, and the procedures were conducted in accordance with the ethical standards stated in the Animal Care and Use Guidelines of KIST. Mice were provided by the animal facility in KIST. Adult male C57BL/6 mice (10 weeks old) were used for the in vivo experiments. Five or six mice were housed in a cage that had a 12:12 light–dark cycle. The temperature and humidity of the animal facility were maintained at 22 ± 2 °C and 50 ± 5%.

### Animal surgery

We provided the full surgical procedure in Supplementary Movie 3. The mice were anesthetized with 4% isoflurane for induction and with 1.5–2% isoflurane during surgeries using an isoflurane vaporizer (SurgiVet Classic T3 vaporizer, Smiths Medical, Inc., Minneapolis, Minnesota, USA). After fixing an anesthetized mouse on a stereotaxic instrument (David Kopf Instruments, USA) (Supplementary Fig. 11a), the hair and scalp were removed (Supplementary Fig. 11b), and this was followed by drilling five sites on the skull, including the target site based on the atlas of Paxinos and Franklin^45^. To firmly secure the neural probe to the skull, we tightened screws in four holes other than the target site (Supplementary Fig. 11c). The neural probe was inserted slowly toward the target sites (Supplementary Fig. 11d, e) that included (1) SN (−3.3; −1.45; −4.3, AP; ML; DV, in millimeters from the bregma), (2) LH (−1.15; −1.05; −5.37, AP; ML; DV, in millimeters from the bregma), and (3) mPFC (+1.9; −0.3; −2.4, AP; ML; DV, in millimeters from the bregma). The probes that were inserted were fixed on the skull with screws and dental cement (Vertex Self Curing, Vertex Dental, Netherlands) (Supplementary Fig. 11f, g). The crown was fixed on the skull with dental cement (Supplementary Fig. 11h). Finally, we confirmed that the pump was operating normally by connecting the wireless module with the battery (Supplementary Fig. 11i). After the surgery, each mouse was housed individually for sufficient recovery to avoid any interferences from other mice. Before performing the in vivo experiments, the mice had a recovery and adaptation period of 1 week. For 4 days, they fully recovered in a home cage under controlled temperature and humidity conditions. During the adaptation period of the next 3 days, the wireless communication module and battery were installed on the heads of the mice so they could adapt to the weight of the system. Also, the handling process was done on the experimenter’s palm for 10 min each day during this period. We conducted in vivo experiments after the recovery and adaptation period.

### Behavior and neural signal analysis

The behavior analysis was performed using two different animal tracking software products, i.e., idTracker^46^ and Optimouse^47^. The behaviors of the mice were expressed as trajectory plots or heatmaps using MATLAB or Python. For the analysis of the neural signals, we applied a custom code to the microcontroller specifically for transmitting spikes. The amplified signals from the signal amplifier chip (RHD2132, Intan Technologies, USA) are transmitted to the microcontroller (TI MSP 430, Texas Instruments, USA) through Serial Peripheral Interface communication. Then, the microcontroller stores the recorded signals that are above the previously set threshold amplitude of 50 μV in the buffer of the microcontroller. Then, the signals stored in the buffer are transmitted to the Bluetooth transceiver (PAN1326B, Panasonic Electronic Components, USA) through serial communication. The Bluetooth transceiver transmits the stored signals to a Bluetooth receiver that is connected to a computer (Supplementary Table 3). The signal data include time periods from –1 to +2 ms based on the threshold amplitude. The signal data were sorted to detect the neural spikes from individual neurons using principal component analysis and the k-means clustering algorithm (https://github.com/akcarsten/spike_sorting). We also compared the timing of the neural spikes recorded from each electrode. When the neural spike timing that was recorded by an electrode coincided with those from the adjacent electrodes, they were regarded as the neural spikes measured from the same neuron^48,49^. Consequently, the firing rates of individual neurons were analyzed.

### Open-field test

To observe whether the weight of the system, including a battery (~4.26 g), constrained the behavior of the mice, we conducted an OFT as was done in previously reported studies^50,51^. Each mouse was placed in an open box (50 × 50 × 40 cm^3^) for 30 min, and its activity was recorded by a camera that was installed above the box. Then, the surgery was done to install the neural probe. After a week of recovery and adaptation, the locomotor activity was recorded using the same mouse but with the whole system, including the battery, mounted on its head. The locomotor activities with and without the system were compared by analyzing both the travel distance and speed using idTracker^46^. In addition, to confirm the behavioral effect of the habituation, we conducted the OFT with normal mice that were not mounted with the system for 5 days. After performing the handling process on a palm for 10 min a day for 3 days, we conduct the OFT with 5 mice for 5 days. In the same way, the locomotor activity was analyzed using idTracker^46^.

### Circling behavior induction experiment

The test of inducing circling behavior by unilateral BIC injection into the SN region of the brain was performed in the same box that was used for OFT. Each test was conducted for a total of 30 min with BIC (0.24 mM) injected for 10 min at the beginning of each test by using a smartphone to control the electrolytic pump wirelessly. The behavior of each mouse was analyzed using the recorded video. We defined circling as a full 360° rotation, and the number of full circles was counted by visual inspection.

### Suppression of feeding behavior

The suppression of feeding behavior by the injection of muscimol into LH was performed in the home cage (20 × 28 × 15 cm^3^) of the mouse without bedding. Prior to the tests, the mice were subjected to caloric restriction for a week. During this period, the mice were given 1.5–2.0 g of food pellets per day to maintain a weight that was 85–90% of their weight as measured immediately after the surgery. The test was conducted with a new food pellet of 3.8–4 g fixed at the center of the home cage. Each test was performed for a total of 20 min while recording a video using a Full HD camera (SPC-B900W, Samsung, South Korea) installed above the home cage. Each mouse underwent two types of tests, i.e., a feeding suppression test with muscimol injection and a control test without muscimol injection. To confirm the appetite throughout the tests, we performed one test per day (with the muscimol injection test first) while maintaining the caloric restriction protocol. The amount of food intake was analyzed by calculating the weight loss of the food pellets during each test. We defined the time spent around the food pellet as the duration of mice bordering the food pellets. The feeding duration was defined as the time the mice spent biting the food pellets. The durations were measured from the recorded video.

### Food competition test

The mice were trained individually for a week to adapt to the protocol related to the provision of food (up to five food pellets were provided in a 10-min period).

The food competition tests were performed in a rectangular field built with an acrylic chamber (40 × 12.5 × 20 cm^3^) in the activity box. There was a hole for providing food pellets on one side of the chamber, and a green LED array was placed on the same side to indicate the provision of food. A long tube (~1 m) for delivering the food pellet was connected with a hole using a glue gun. A Full HD camera also was installed above the chamber to record the competition for food between the pairs of mice. In the training and food competition test, we manually turned the green LED on and off using a rocker switch. Also, the food pellet was injected manually into the tube. Two persons performed the food competition test because the switching of LED status, delivery of food pellets, and the operation of the computer were done manually.

We paired mice of the same age that were grown in different cages to prevent unilateral winning in the food competition. Within a pair of mice, one mouse had a probe inserted in mPFC with a green LED attached to the system, and the other mouse had a probe inserted in LH with a red LED attached to the system. For the food competition tests, we used small food pellets that weighed 20 mg (F0163, Bio-Serv, USA), and the mice were adapted to the food pellets during the 1-week caloric restriction period. Also, in this period, they were trained individually for the experimental protocol. Individual training was performed for 10 min in the same chamber as the food competition test. The green LED was turned on, and food pellets were provided 5 times every 2 min. Because Bluetooth communication only permits one-to-one connection, two laptops were utilized for recording the neural signal from each mouse. The time of the two laptops was synchronized to Korean standard time using the UTCk 3.1 software provided by the Korea Research Institute of Standards and Science.

Each test of the competition for food was performed for a total of 20 min. To conduct an unbiased competition for food, we defined the half of the field that was opposite the side where the food was provided as the start zone, and a food pellet was provided once every 2 min only when both mice were in the start zone. The food pellet was provided only once, even though both mice were in the starting zone several times within the 2 min. However, unless both mice were in the starting zone within 2 min, the food pellet was not provided, which was defined as a passed trial. In addition, a competition trial was defined as the situation in which two mice competed for the food pellet, and a non-competition trial was defined as the situation in which only one mouse approached the food and ate the food pellet without competition. And, the beginning of the trial was defined as the situation in which the food pellet was provided with the LED cue, and the end of the trial was defined as the situation when the mice finished eating the food pellet, respectively. In summary, the food competition test was conducted for 20 min, and when the food-providing condition was met, one food pellet was provided manually every 2 min. Thus, a maximum of ten trials was conducted per test. Using the three pairs of mice, we conducted the food competition tests twice a day for 4 days. In the first test, we did not inject muscimol into the LH of the red mouse. After resting for at least 2 h, the second test was conducted with the muscimol injection into the LH of the red mouse. By reviewing the recorded video, we were able to analyze the time at which a mouse reached for a food pellet that had been provided, the presence of competition, and the winning history, all of which were analyzed through visual inspection.

### Statistical analysis

The statistical analyses were performed using MATLAB (MathWorks, USA), Python (Python Software Foundation, USA), and GraphPad Prism (GraphPad, USA). The normality of distributions from all statistically analyzed data was assessed by GraphPad Prism. If normality was achieved, the statistical analyses were assessed by parametric analysis (e.g., the two-tailed paired/unpaired *t*-test or one-way ANOVA with Tukey’s multiple comparisons test). If normality was not achieved, the statistical analyses were assessed by non-parametric analysis (e.g., Mann–Whiney test, Wilcoxon matched-pairs signed rank test, or Friedman test with Dunn’s multiple comparisons test). Also, all of the statistical analyses were based on independent samples. The statistical analysis method used for each set of data is included in the legend of each figure. Also, the detailed results of the statistical analysis are included in a single Excel file as additional supplementary data.

### Reporting summary

Further information on research design is available in the Nature Research Reporting Summary linked to this article.

## Supplementary information

Supplementary Information

Description of Additional Supplementary Files

Supplementary Movie 1

Supplementary Movie 2

Supplementary Movie 3

Supplementary Movie 4

Supplementary Movie 5

Supplementary Movie 6

Supplementary Movie 7

Supplementary Movie 8

Supplementary Movie 9

Supplementary Movie 10

Supplementary Movie 11

Supplementary Movie 12

Reporting Summary

## Data Availability

The authors declare that all data supporting the findings of this study are available within the article and its supplementary information files. Source Data are provided with this paper.

## Source data

Source Data

## Code availability

The custom codes applied in the wireless communication module and used for data extraction, and custom software for neural signal recording are freely available at https://github.com/HyogeunShin/Wireless-System_NatComm22 (10.5281/zenodo.7032078). The custom code applied in the wireless communication module was written in C language. Also, the custom code used for data extraction was written in MATLAB.
