# Supplementary material for: Neural probe system for behavioral neuropharmacology by bi-directional wireless drug delivery and electrophysiology in socially interacting mice
Source: Nat Commun. 2022 Sep 21;13:5521. doi: 10.1038/s41467-022-33296-8 (PMC9492903; doi:10.1038/s41467-022-33296-8)
Supplement: Supplementary file 1 — Supplementary Information [file 41467_2022_33296_MOESM1_ESM.pdf]

Supplementary Information:

Neural probe system for behavioral neuropharmacology by bi-directional wireless drug delivery and electrophysiology in socially interacting mice

Yoon and Shin et al.

	Proposed system	[1]Dagdeviren et al., 2018	[2]Cobo et al., 2016	[3]Zhang and Castro et al., 2019	[4]Qazi et al., 2019
Capability	**Electrical recording and drug delivery**	Electrical recording and drug delivery	Drug delivery	Drug delivery and optogenetics	Drug delivery and optogenetics
Operation (Wireless or Wired)	**Wireless**	Wired (Wireless in case of the pump)	Wireless	Wireless	Wireless
Type	**Electrochemical**	Mechanical	Electrochemical	Electrochemical	Thermal
Integration of drug reservoir	**Yes**	Yes	Yes	Yes	Yes
Volume of drug reservoir	**50 μL**	900 μL	276 μL (96 μL of dead volume)	0.25 μL per chamber (4 chambers)	0.5 μL per chamber (4 chambers)
Injection volume control	**Controllable**	Flexible	Flexible	Fixed (0.25 μL)	Fixed (0.5 μL)
Repetitive drug delivery	**Yes**	Yes	Yes	Partially yes (Only four times)	Partially yes (Only four times)
Backflow	**Not observed**	Not observed	Maximum of 10% against infused volume	Not observed	Not observed
Sound noise	**No**	Yes	No	No	No
Drug reservoir use	**Refillable reservoir**	Refillable reservoir	Refillable reservoir	Refillable reservoir	Replaceable cartridge
Temperature change during operation / Possibility of drug degradation	**Unchangeable / No**	Unchangeable / No	Unchangeable / No	Unchangeable / No	Changeable / Yes
Size (mm^3)	**~2,000 mm^3**	5,356 mm^3	2,430 mm^3	314 mm^3	1,260 mm^3
Weight (g)	**4.6 g**	6.6 g	3 g	0.29 g	2 g
Power consumption of the pump (mW)	**5 mW**	-	1 mW	1-3 mW	> 100 mW

Supplementary Table 1: A summary of our system`s performance and comparison with previously developed systems with drug delivery capability.

	**Proposed system**	**Thomas wireless system (TWS)<br>(Thomas RECORDING)**	**W2100-HS8-ES2<br>(Multichannel systems)**
Number of<br>recording channels	**16-ch**	4-ch	8-ch
Number of<br>stimulation channels	**2-ch**	1-ch	2-ch
Sampling rate (Resolution)	**8 kHz (16-bit)**	20 kHz (12-bit)	25 kHz (16-bit)
Transmission protocol	**Bluetooth 4.0**	-	-
Number of devices<br>that can be recorded<br>simultaneously	**6<br>[5](simultaneously six mice per the cage)**	-	-
Transmission distance (m)	**~ 50 m**	~ 5 m	~ 5 m
Size (mm^3)	**1,350 mm^3**	6,864 mm^3	1,800 mm^3
Weight without battery (g)	**2.36 g**	4 g	3.8 g
Weight with battery (g)	**3.3 g**	11.2 g	5.4 g
Power consumption (mW)	**79 mW<br>(~80 min using 30 mAh Li-Po battery)**	888 mW<br>(1 hours using 240 mAh lithium battery)	166 mW<br>(40 min using 30 mAh Li-Po battery)

Supplementary Table 2: A summary of the bidirectional wireless system`s performance and comparison with commercially available systems.

	Minimum specifications	Recommended specifications
Processor	Intel i5-8265U	Intel i7-8565U
Operating system	Windows 10 (64-bit)	Windows 10 (64-bit)
Memory	8 GB of RAM (DDR4)	16 GB of RAM (DDR4)
GPU	Not required	Not required
Storage	8 GB of available hard-disk space	8 GB of available hard-disk space

Supplementary Table 3: Required hardware specification to record neural signals using the bi-directional wireless communication module.

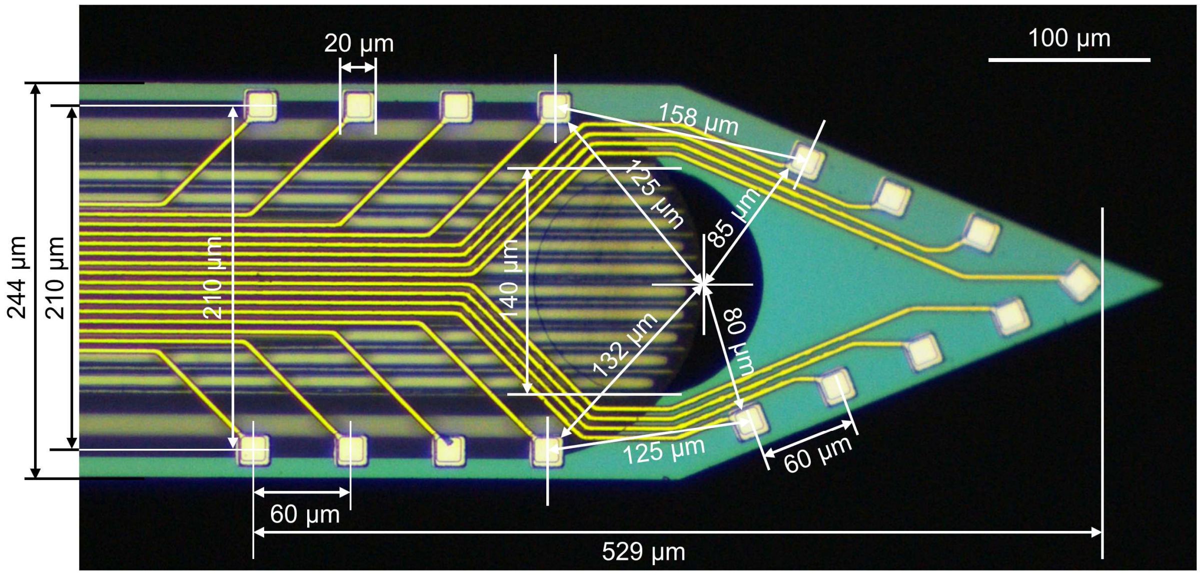

Supplementary Fig. 1: Detailed dimensions for the fabricated neural probe.

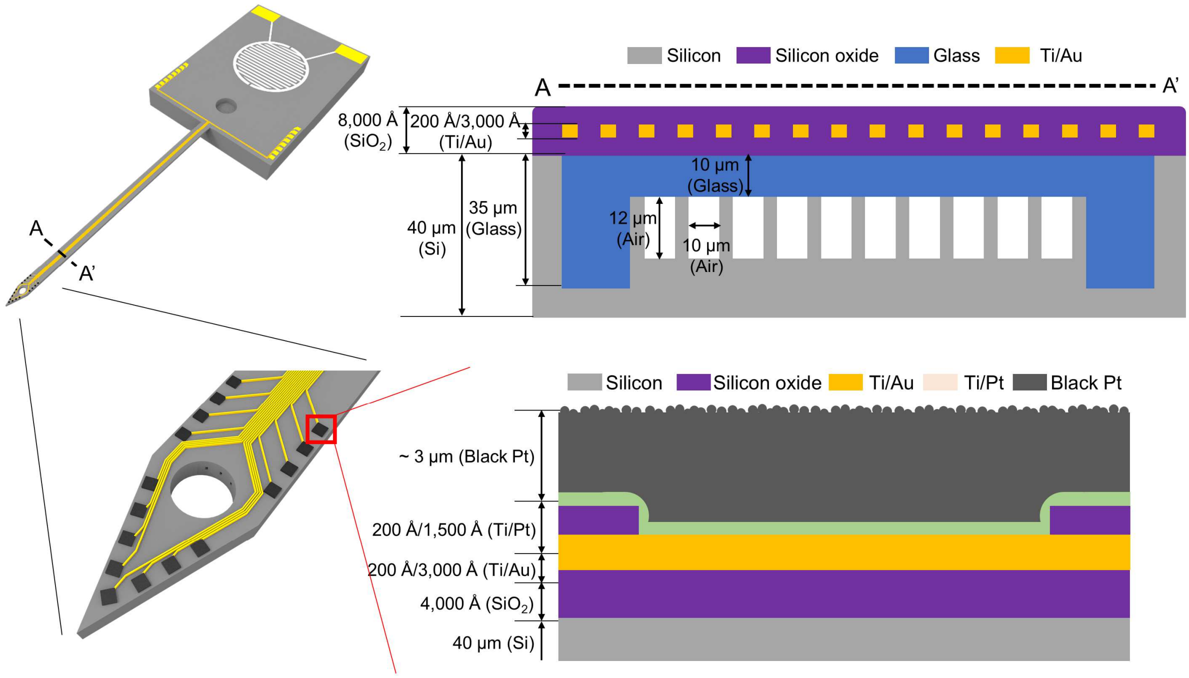

Supplementary Fig. 2: Cross-sectional schematic for showing the detailed thickness and material information of the fabricated probe's shank with electrodes.

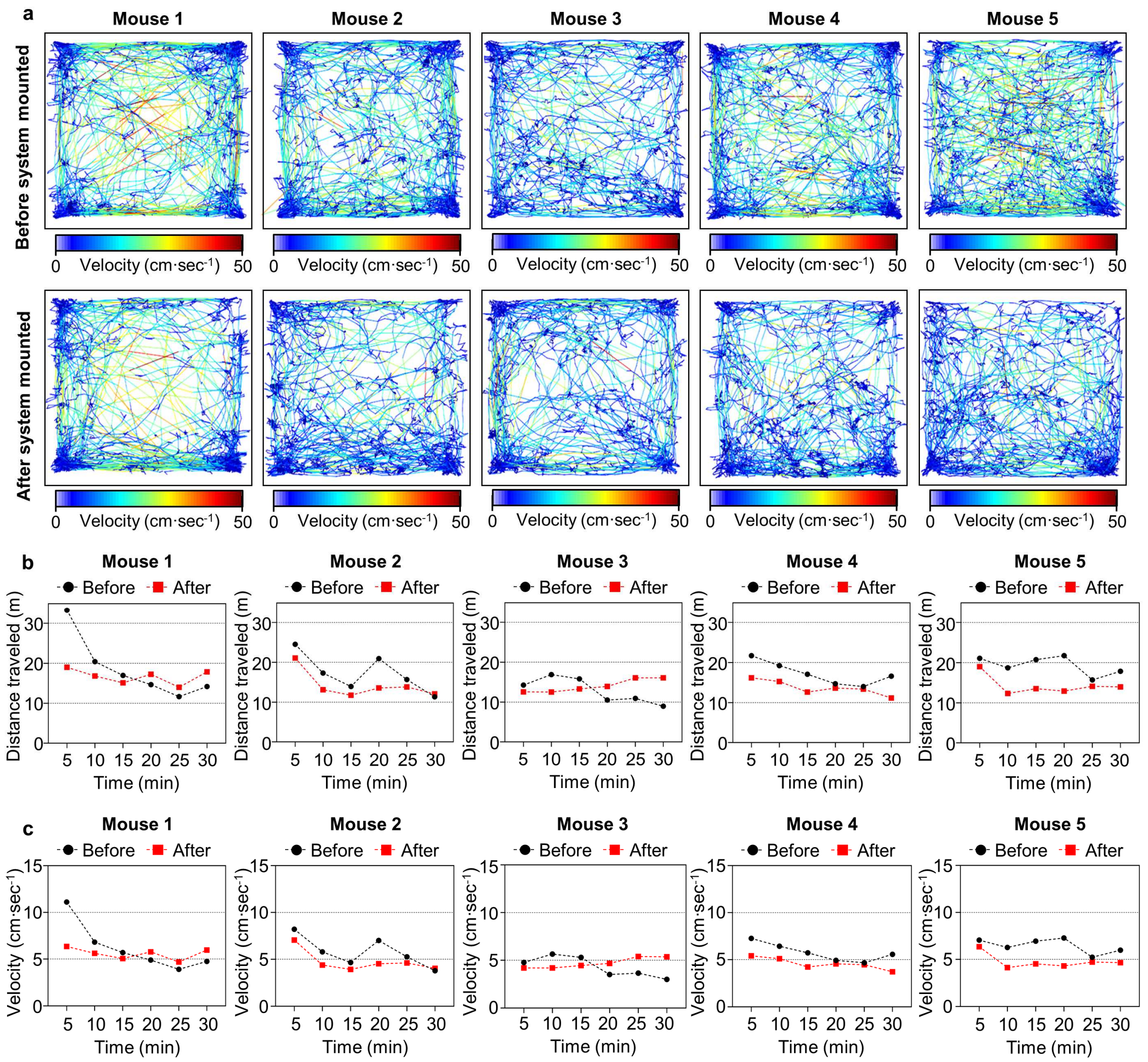

Supplementary Fig. 3: Changes in locomotor activity before and after mounting the system. a Trajectories with velocity heatmap of each mouse before and after mounting the system during 30 min. **b** Distance traveled of each mouse in 5 minutes intervals. **c** Mean velocity of each mouse in 5 minutes intervals.

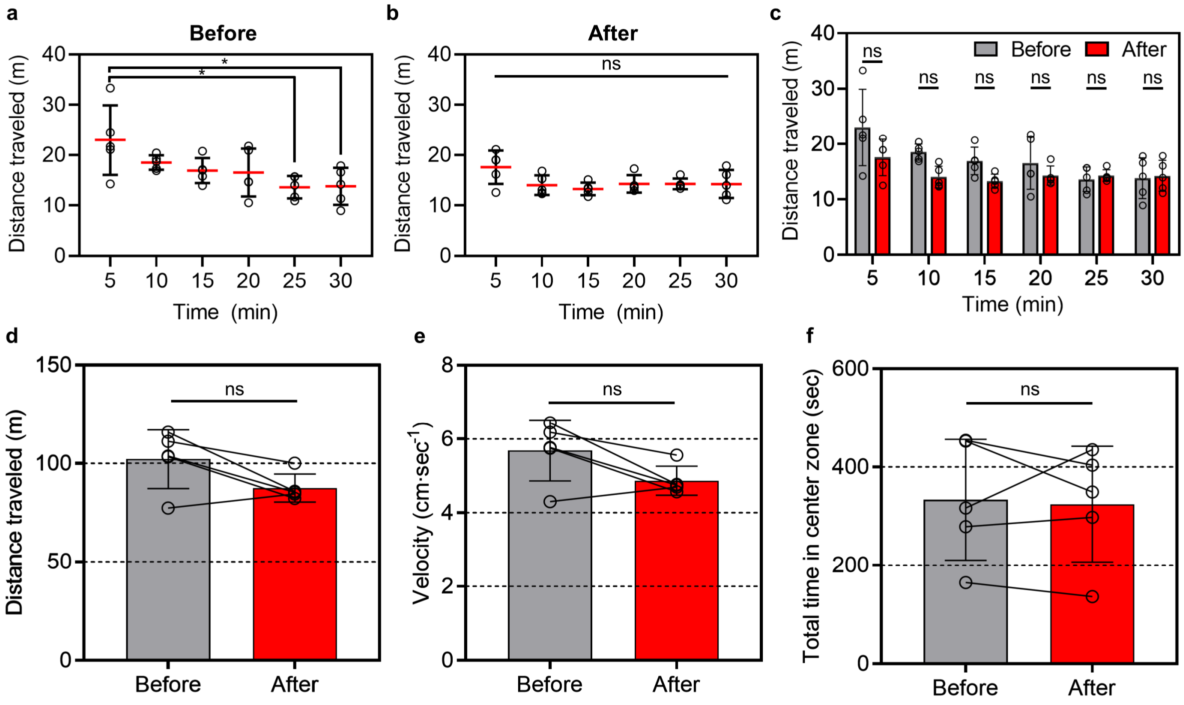

Supplementary Fig. 4: Effect of neural probe system on general locomotor activities. a Comparison of the distance traveled in 5 minutes intervals before mounting the system on the head of the mice (5 min vs 25 min: p=0.03519; 5 min vs 30 min: p=0.03519). **b** Comparison of the distance traveled in 5 minutes intervals before mounting the system on the head of the mice. **c** Comparison of the distance traveled in 5 minutes intervals between before and after mounting the system on the head of the mice (5 min: p=0.06250; 10 min: p=0.06250; 15 min: p=0.06250; 20 min: p=0.62500; 25 min: p=0.81250; 30 min: p>0.99999). **d** Comparison of the distance traveled before and after mounting the system on the head of the mice (p=0.12500). **e** Comparison of velocity between before and after mounting the system on the head of the mice (p=0.12500). **f** Comparison of total time in the center zone between before and after mounting the system on the head of the mice (p=0.81250). Data are presented as mean values +/− s.d. with individual data points (white circle: n = 5 for all data, n is the number of mice). Statistical analyses were performed by Friedman test with Dunn's multiple comparisons test in Supplementary Fig. 4a-b and by Wilcoxon matched-pairs signed rank test in Supplementary Fig. 4c-f. p<0.05 was considered significant. * p<0.05. ns: no statistical significance.

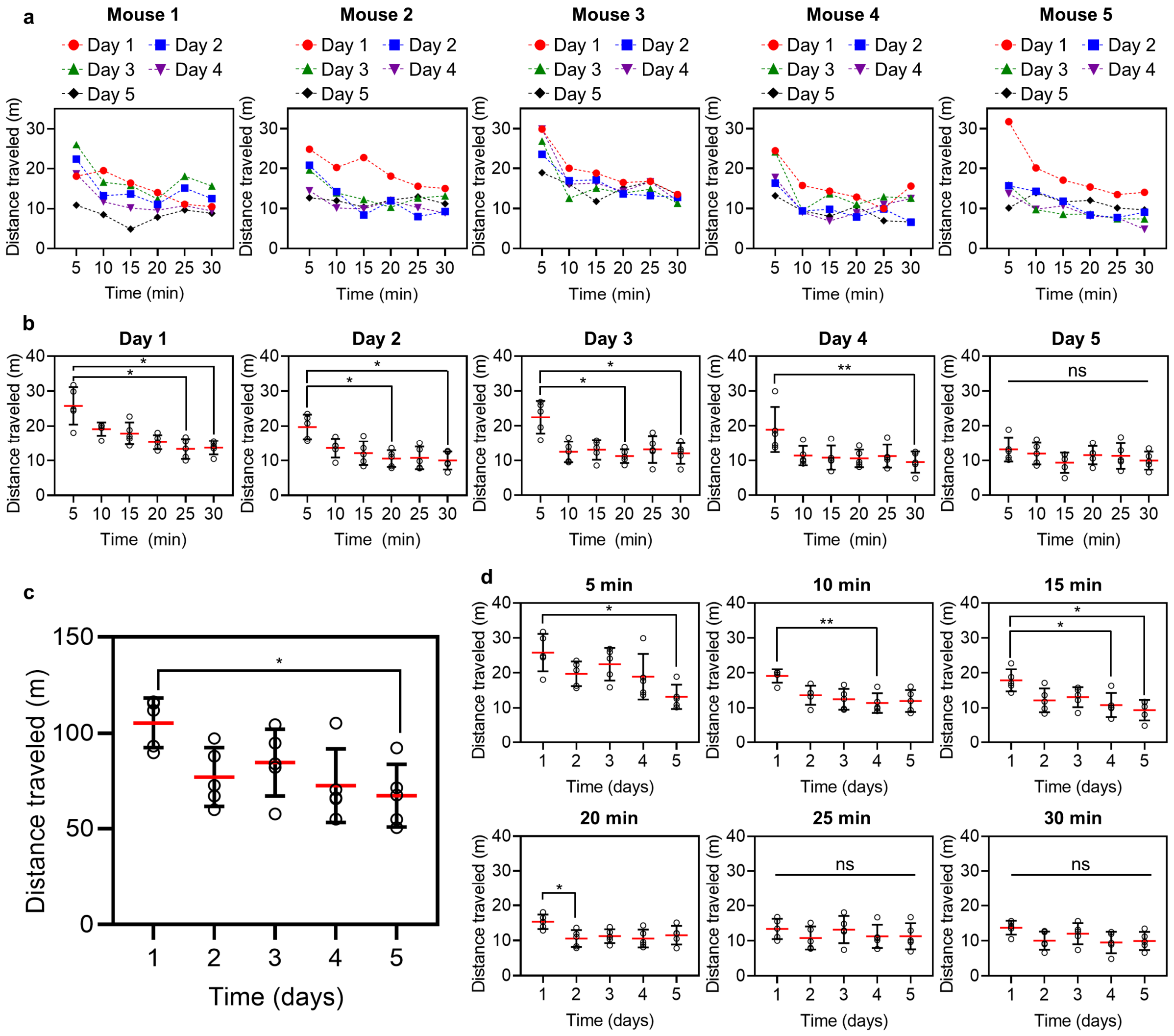

Supplementary Fig. 5: Changes in locomotor activity according to repetitive tests in normal mice not mounted with the system. a Distance traveled of each mouse in a day interval. **b** Comparison of the distance traveled in 5 minutes intervals from Day 1 to Day 5 (**Day 1**: p=0.01085 (5 min vs 25 min), p=0.01085 (5 min vs 30 min); **Day 2**: p=0.03519 (5 min vs 20 min), p=0.01085 (5 min vs 30 min); **Day 3**: p=0.01980 (5 min vs 20 min), p=0.01980 (5 min vs 30 min); **Day 4**: p=0.00579 (5 min vs 30 min)). **c** Comparison of the distance traveled between days (day 1 vs day 5: p=0.02700). **d** Comparison of the distance traveled between days in 5 minutes intervals (**5 min**: p=0.01374 (day 1 vs day 5); **10 min**: p=0.09322 (day 1 vs day 4); **15 min**: p=0.02700 (day 1 vs day 4), p=0.02700 (day 1 vs day 5); **20 min**: p=0.01374 (day 1 vs day 2)). Data are presented as mean values +/− s.d. with individual data points (white circle: n = 5 for all data, n is the number of mice). All statistical analyses were performed by Friedman test with Dunn's multiple comparisons test. p<0.05 was considered significant. * p<0.05, ** p< 0.01. ns: no statistical significance.

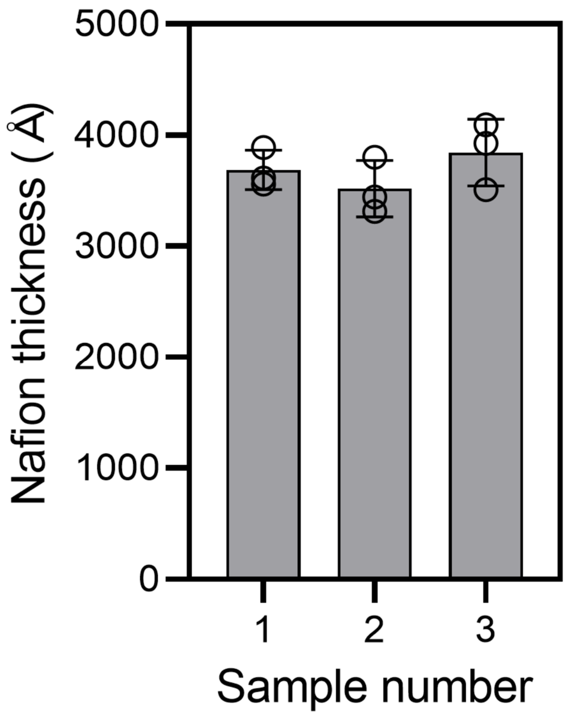

Supplementary Fig. 6: The thickness of Nafion at three points from three samples. Data are presented as mean values +/− s.d. with individual data points (white circle: n = 3 for all data, n is the number of measurements at different locations).

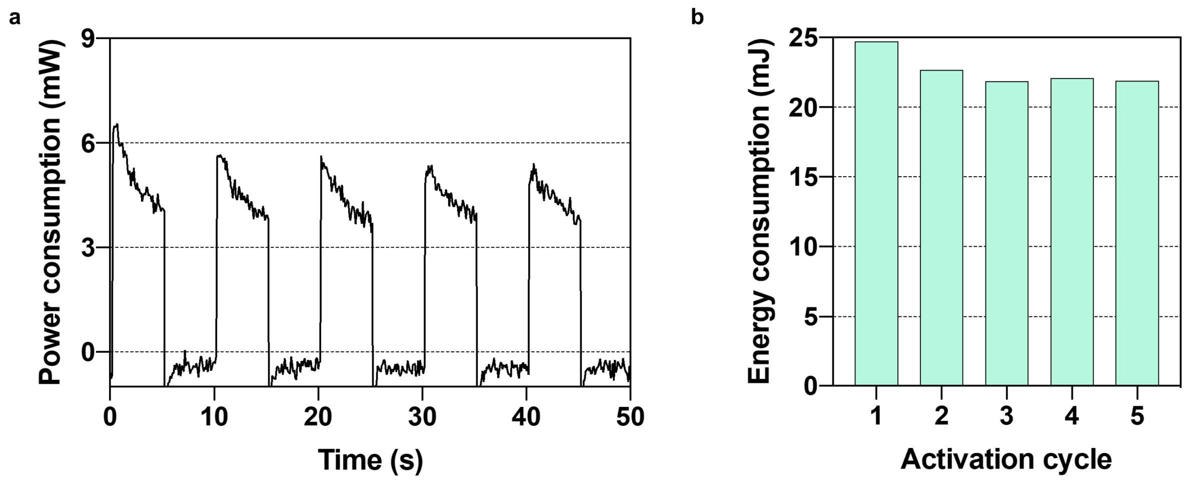

Supplementary Fig. 7: Power consumption of the miniaturized electrolytic pump. a Power consumption over time by activating the pump with five cycles (5 s on and 5 s off over a 10 s period). **b** Energy consumption per cycle by activating the pump with five cycles (5 s on and 5 s off over a 10 s period).

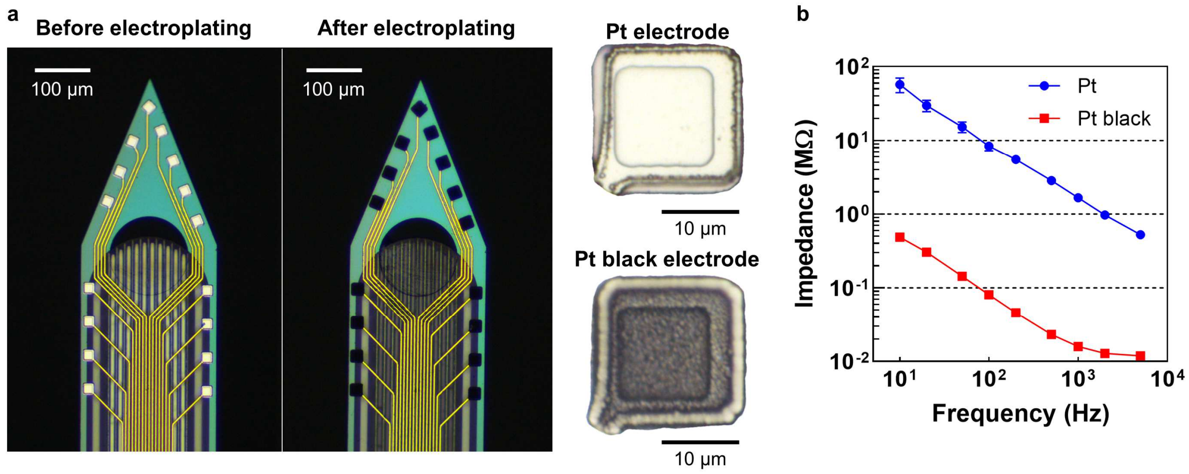

Supplementary Fig. 8: Electrical characterization of the neural probe. a Photographs showing the Pt and Pt black electrodes before and after Pt black electroplating, respectively. **b** The electrical impedance of the 16 microelectrodes before and after electroplating of Pt black. Data are presented as mean values +/− s.d. (n = 16 for all data, n is the number of electrodes).

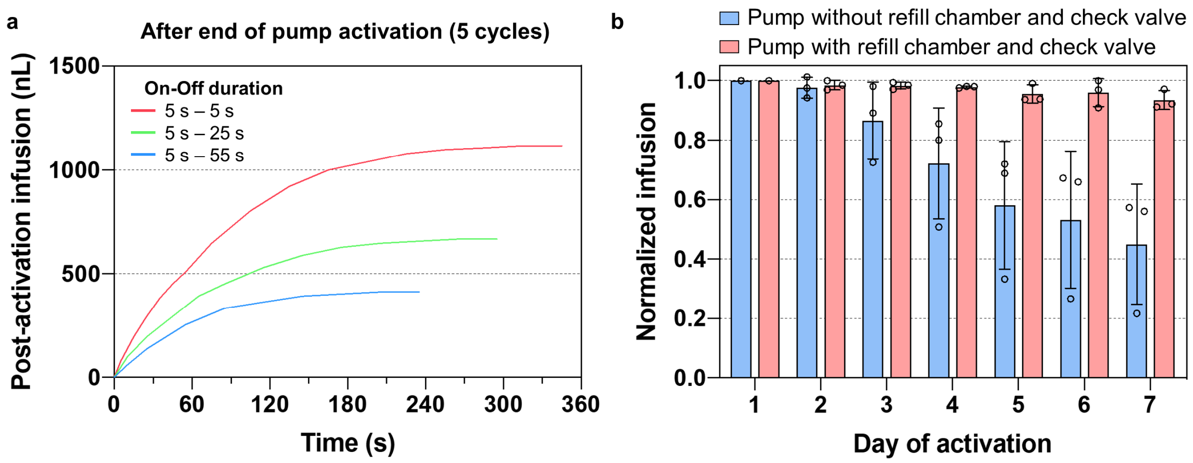

Supplementary Fig. 9: Supplementary features of the miniaturized electrolytic pump. a Infused volume over time after turning off the fifth pump activation cycle. **b** Comparison of the daily infusion performance between the pump without refill chamber and check valve and the pump with refill chamber and check valve. Data are presented as mean values +/− s.d. with individual data points (white circle: n = 3 for all data, n is the number of samples).

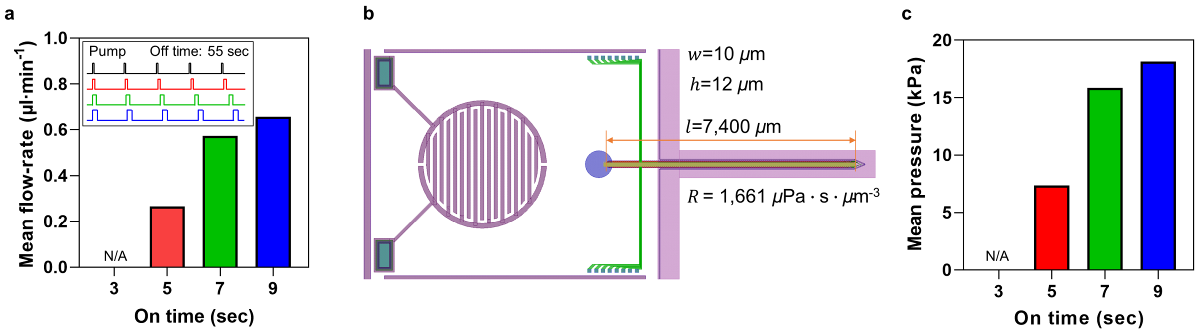

Supplementary Fig. 10: Mean flow-rate and pressure according to activation time. a Measured mean flow-rate according to activation time (3 sec, 5 sec, 7 sec, and 9 sec) (5 cycles; 55 sec off). The sketch inset in the graphs shows the on and off times of the pump over a whole period. **b** Calculated hydraulic resistance of the probe through width (*w*), height (*h*), and length (*l*) of the 10-microfluidic channels. *R* is the fluidic resistance of the probe. **c** Calculated mean pressure based on the measured flow rate according to activation time (3 sec, 5 sec, 7 sec, and 9 sec) (5 cycles; 55 sec off).

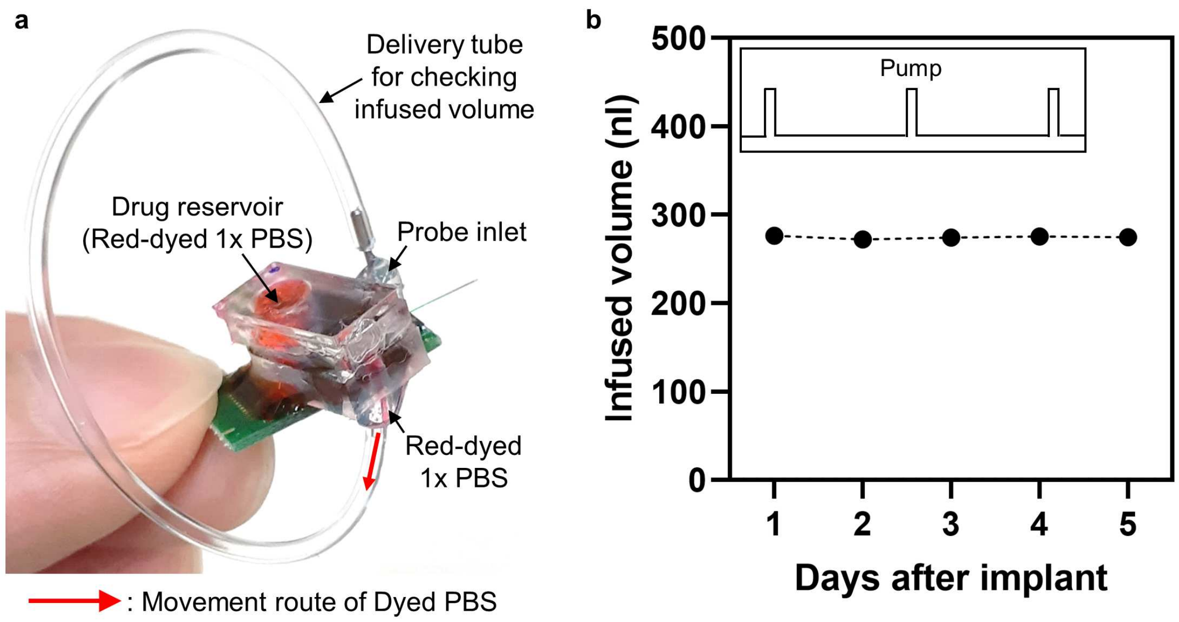

Supplementary Fig. 11: Monitoring of infused volume during 5 days in the mouse brain. a Packaged device for measuring infused volume in the mouse brain. The movement distance of red-dyed 1x PBS in the delivery tube was measured under the same operating conditions (3 cycles; 5 s on and 55 s off over a 60 s period) in the anesthetized mouse. The infused volume was calculated based on the movement distance of red-dyed 1x PBS. **b** Infused volume of PBS solution into the brain for 5 days. The sketch inset in the graphs shows the on and off times of the pump over a whole period.

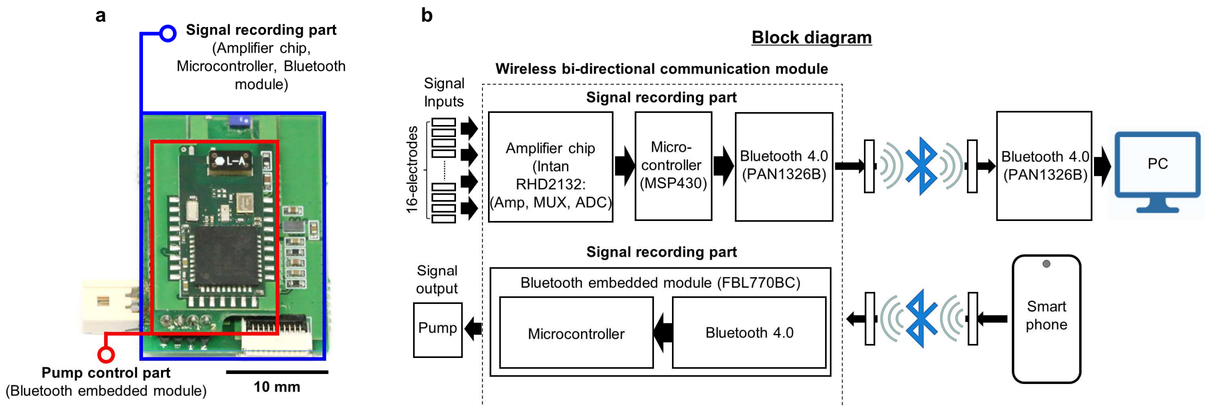

Supplementary Fig. 12: Summary of the bi-directional wireless communication module. a The picture shows a bi-directional wireless communication module that consists of signal recording and pump control parts. **b** Block diagram of the bi-directional wireless communication module.

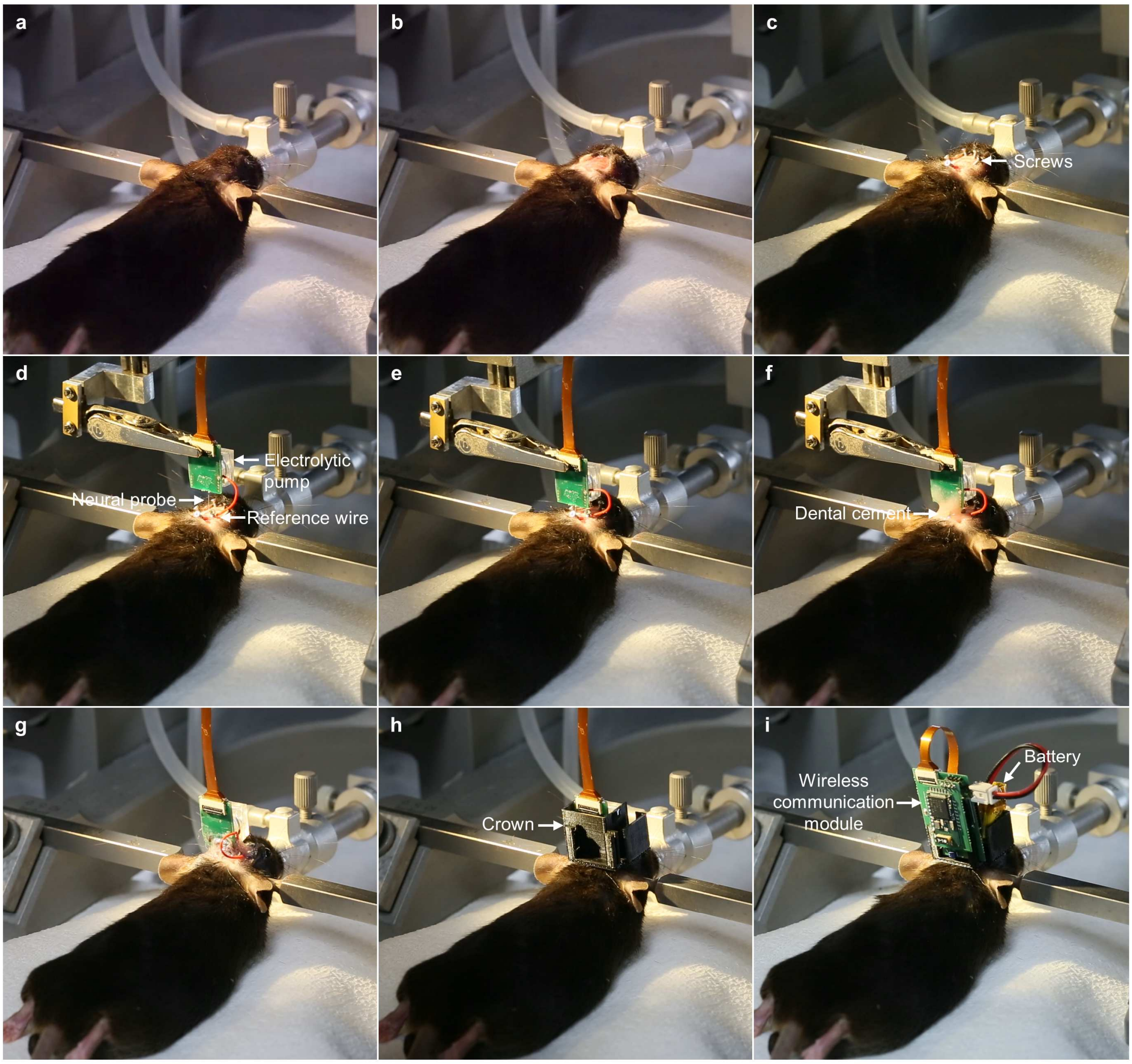

Supplementary Fig. 13: Surgical process. **a** Fixation of the mouse's head in the stereotaxic instrument. **b** Removal of the mouse's hair and scalp. **c** Drilling the holes into the skull and tightening the set screws into the holes. **d** Movement of the neural probe over the mouse's head and placement of the reference wire on the contralateral cerebral surface. **e** Insertion of the neural probe into the brain. **f-g** Fixation of the neural probe to the skull and set screw using the dental cement. **h** Fixation of the crown to the mouse's head using dental cement. **i** Connection of the wireless system with the battery for verifying signal recording and pump operation.

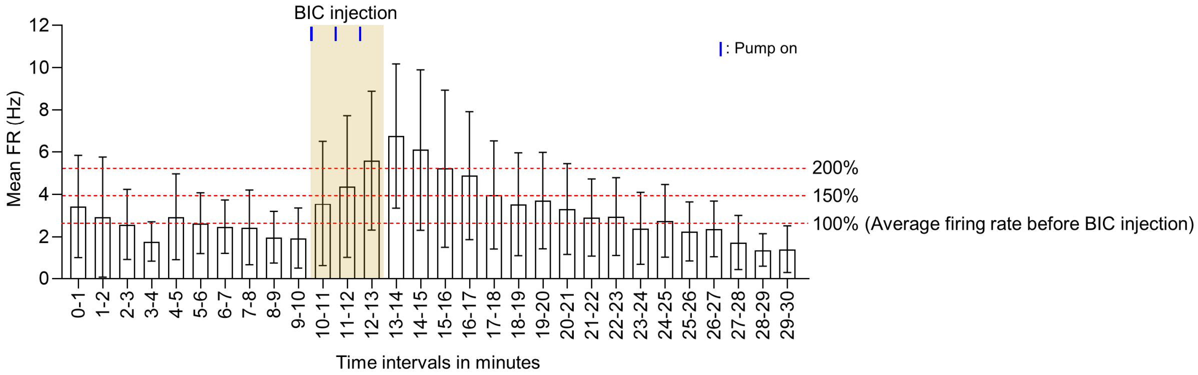

Supplementary Fig. 14: The changes in the firing rate of SN neurons (n = 6 where n is the number of neurons) before and after BIC injection in minutes scale. Data are presented as mean values +/− s.d.

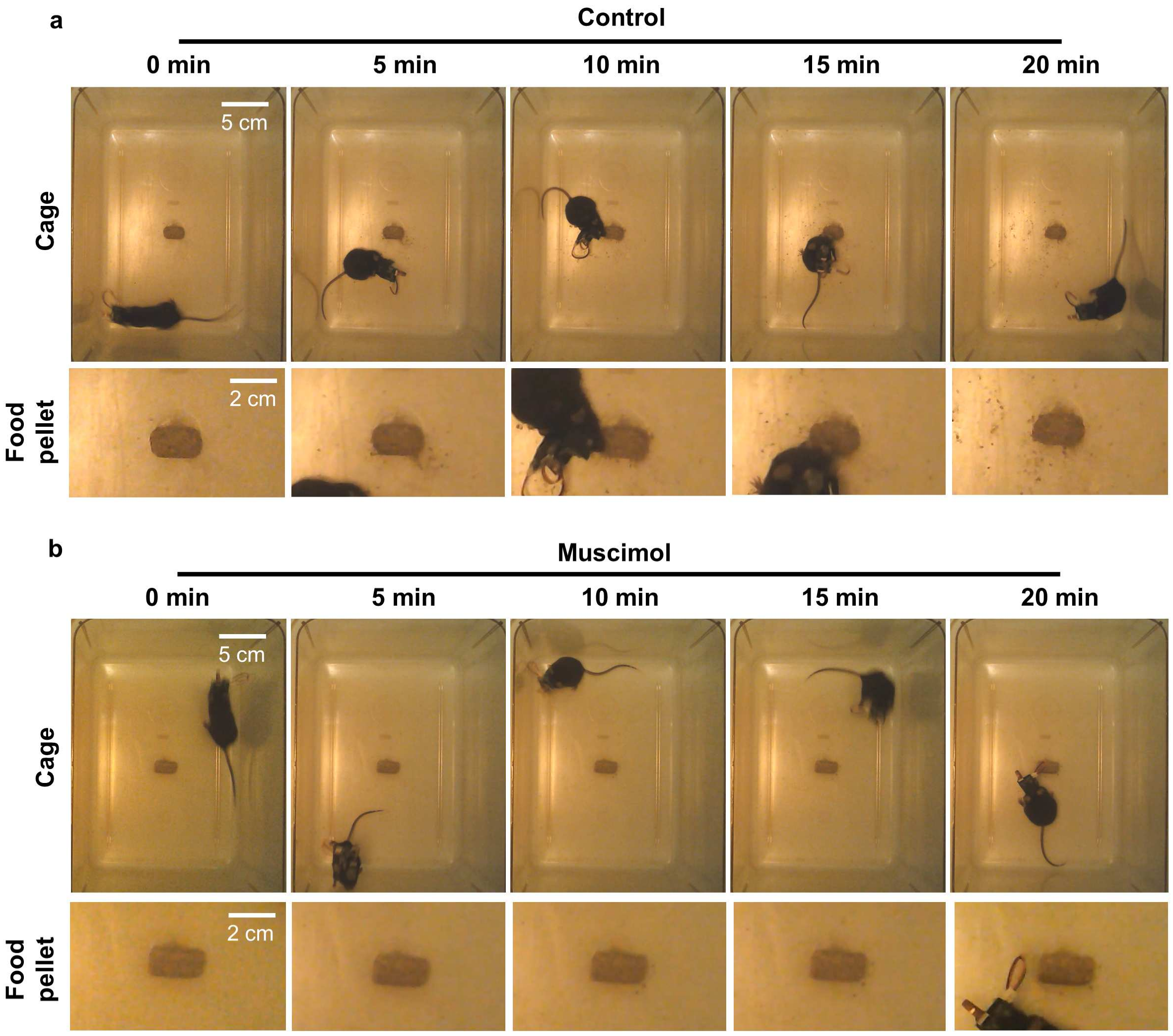

Supplementary Fig. 15: Comparison of feeding between the control mouse and the muscimol injected mouse. a-b Successive pictures showing the feeding behaviour and the size of consumed food pellet over time in **(a)** the control experiment and in **(b)** an experiment with the muscimol injection.

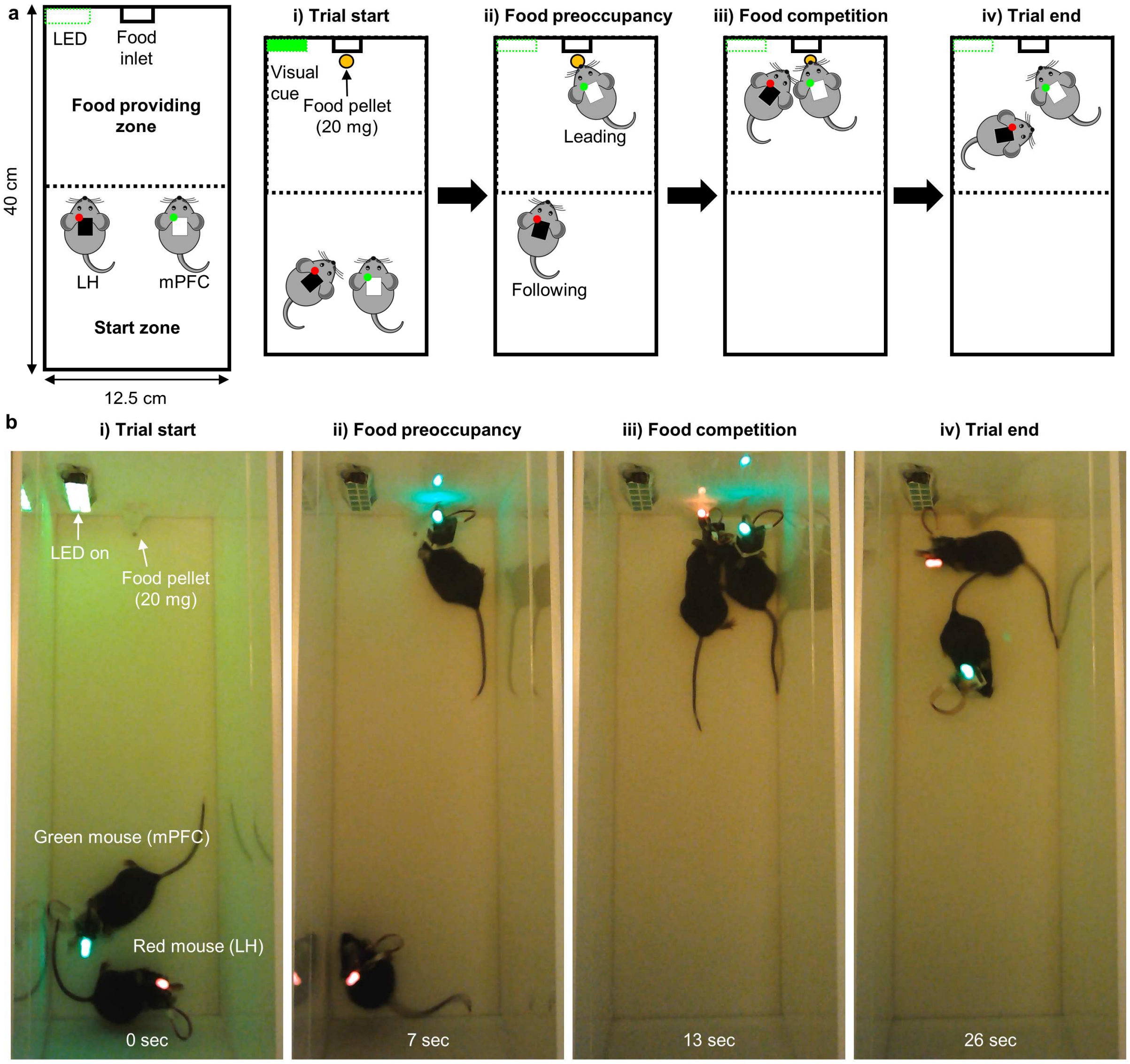

Supplementary Fig. 16: Protocol of the food competition experiment. a Schematic diagrams showing the experimental setup and the food competition procedure: i) Start of a trial when both mice are located in the start zone, ii) Food preoccupancy by a leading mouse that arrives first, iii) Competition for food after the arrival of the following mouse, and iv) Trial end after consuming the food pellet and separation of the two mice. **b** Successive pictures showing each step of the food competition test.

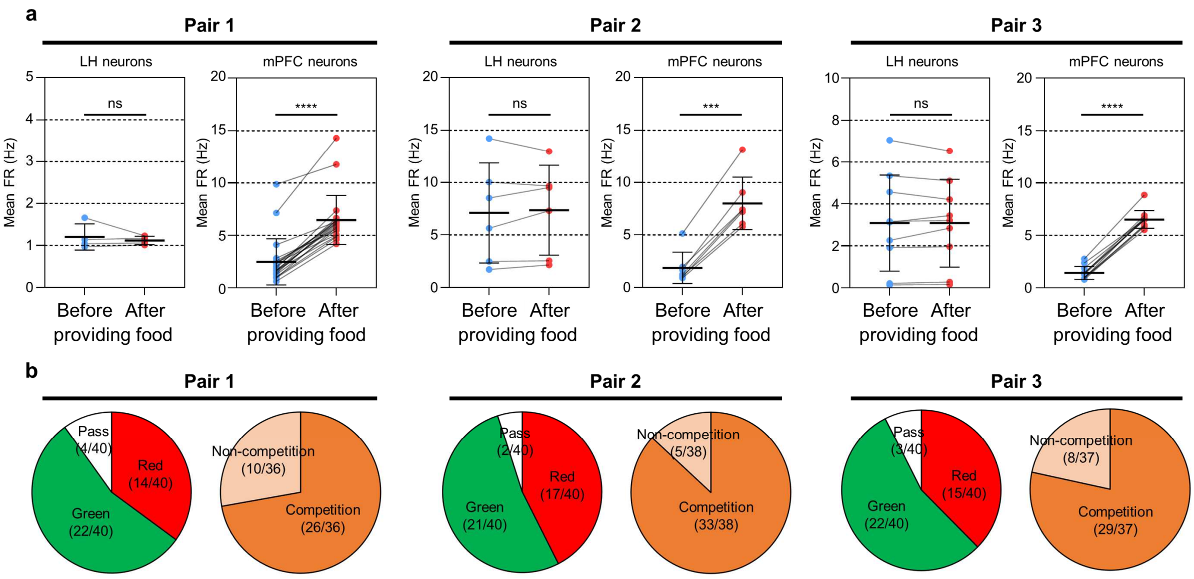

Supplementary Fig. 17: Neural activities of LH and mPFC neurons during food competitions. a Comparison of mean firing rates between the before trial (i.e., before providing the food pellet) and the during trial (i.e., after providing the food pellet) from LH and mPFC neurons of each mouse pair (**Pair 1-LH mouse**: n=4; p=0.88571; **Pair 1-mPFC mouse**: n=21; p<0.00001; **Pair 2-LH mouse**: n=6; p=0.93723; **Pair 2-mPFC mouse**: n=7; p=0.00058; **Pair 3-LH mouse**: n=9; p=0.86331; **Pair 3-mPFC mouse**: n=12; p<0.00001). **b** Winning ratio and incidence of competition of each mouse pair throughout the food competition tests (4 tests per each pair). Data are presented as mean values +/− s.d. with individual data points. n is the number of neurons. All statistical analyses were performed by Mann Whitney test. p<0.05 was considered significant. *** p< 0.001, **** p<0.0001. ns: no statistical significance.

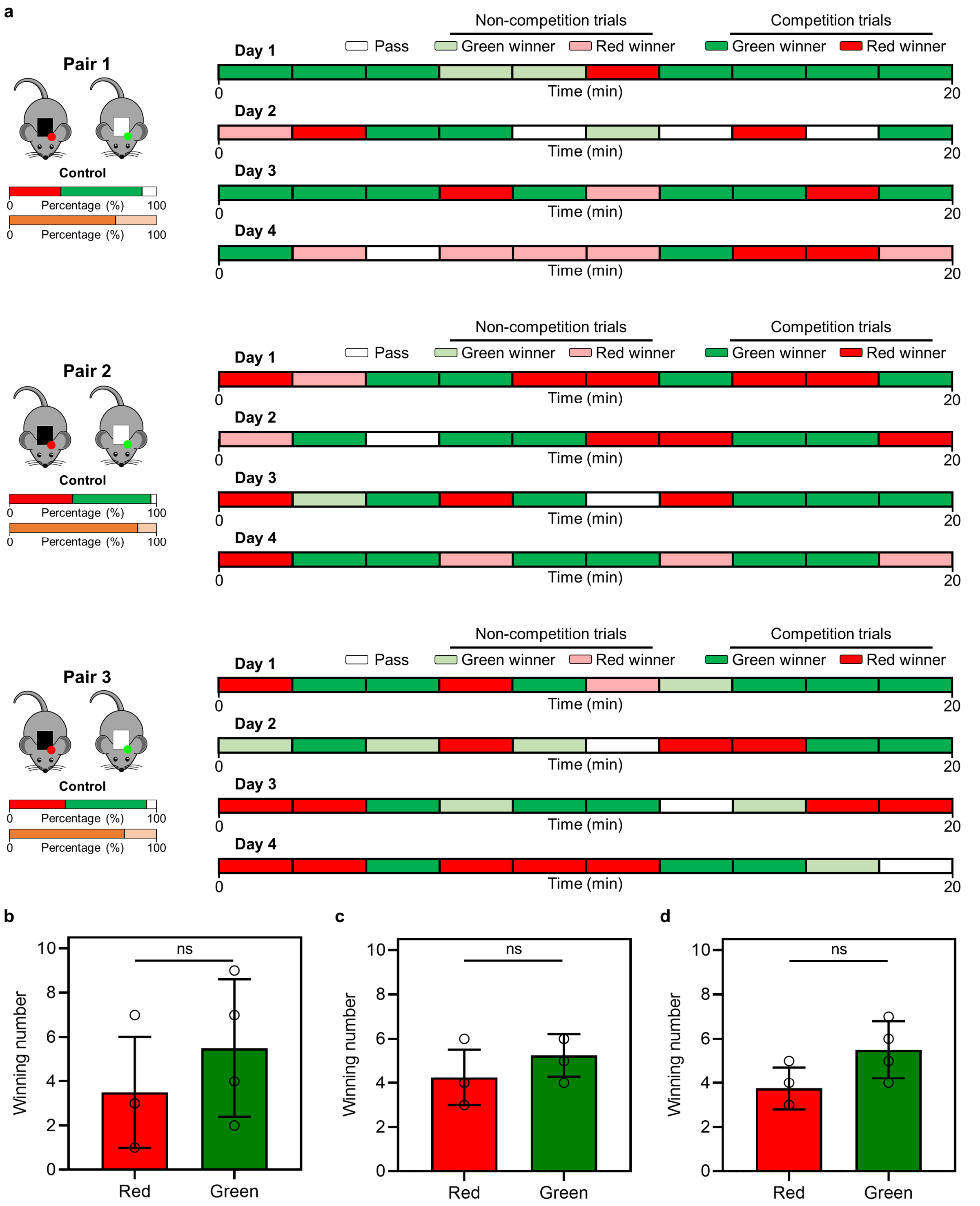

Supplementary Fig. 18: Detailed winning history of each mouse pair. a Chart showing all dates in each pair. **b-d** Bar graphs showing a comparison of the number of wins between two mice for (**b**) pair 1 (p=0.42860), (**c**) pair 2 (p=0.40000), and (**d**) pair 3 (p=0.14290). Here, n is the number of tests; Data are presented as mean values +/− s.d. with individual data points (white circle: n = 4 for all data, n is the number of the test). All statistical analyses were performed by Mann Whitney test. ns: no statistical significance.

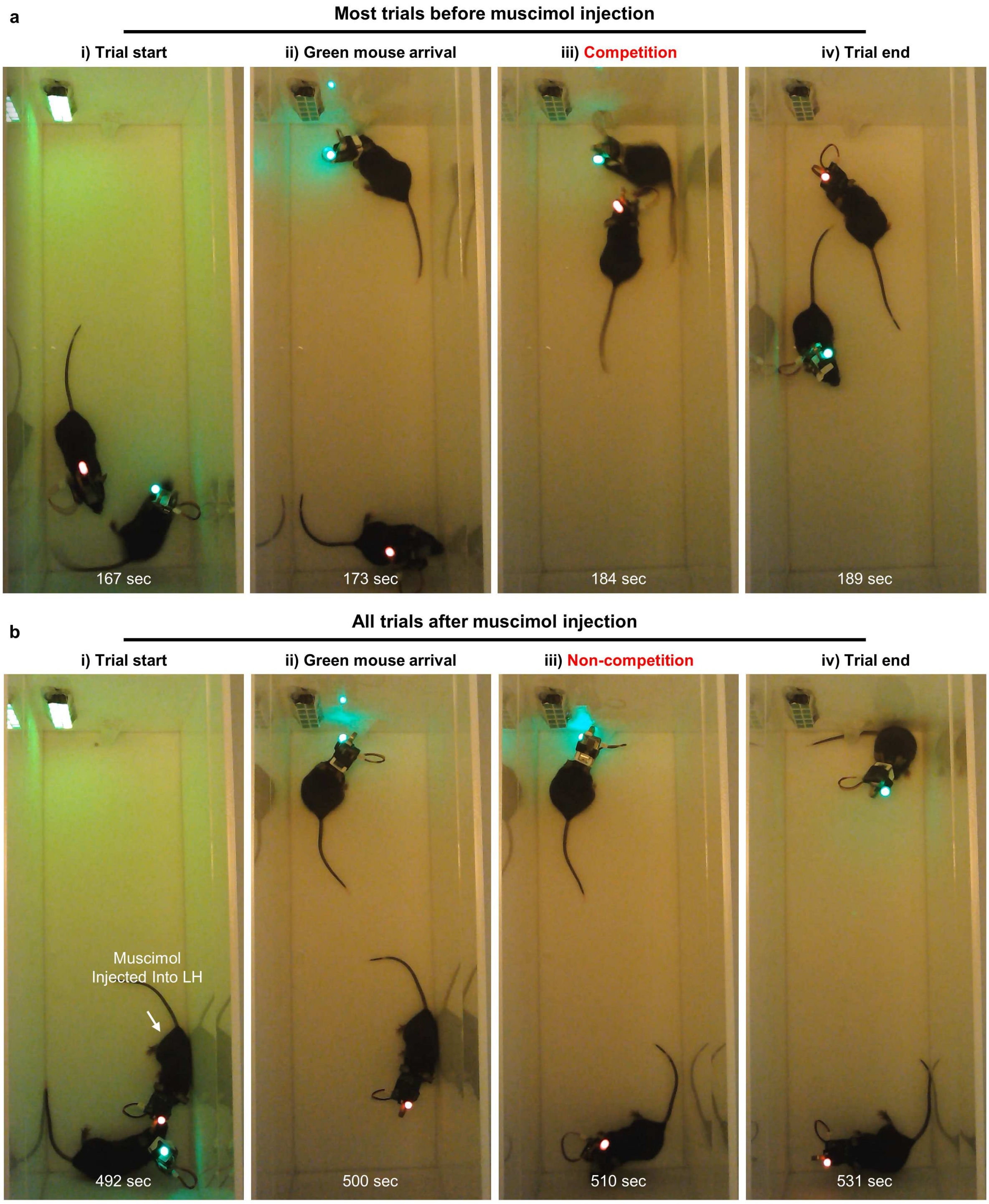

Supplementary Fig. 19: Comparison of food competition between the trials before and after muscimol injection. a-b Successive pictures showing each step for food competition **(a)** before muscimol injection and **(b)** after the muscimol injection.

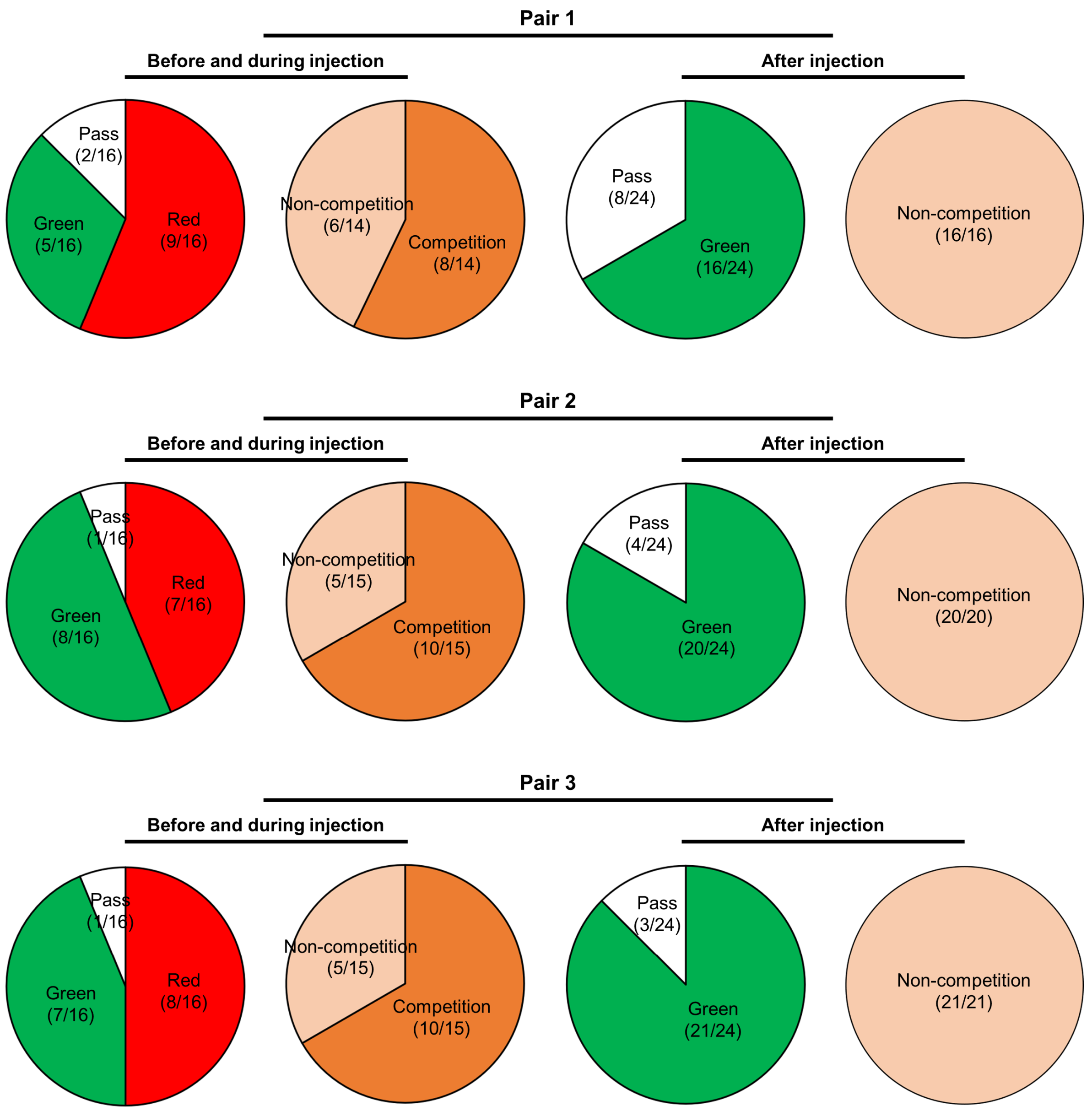

Supplementary Fig. 20: Winning ratio and incidence of competition of each mouse pair before and during muscimol injection and after muscimol injection throughout the food competition test that included muscimol injection (4 tests per pair).

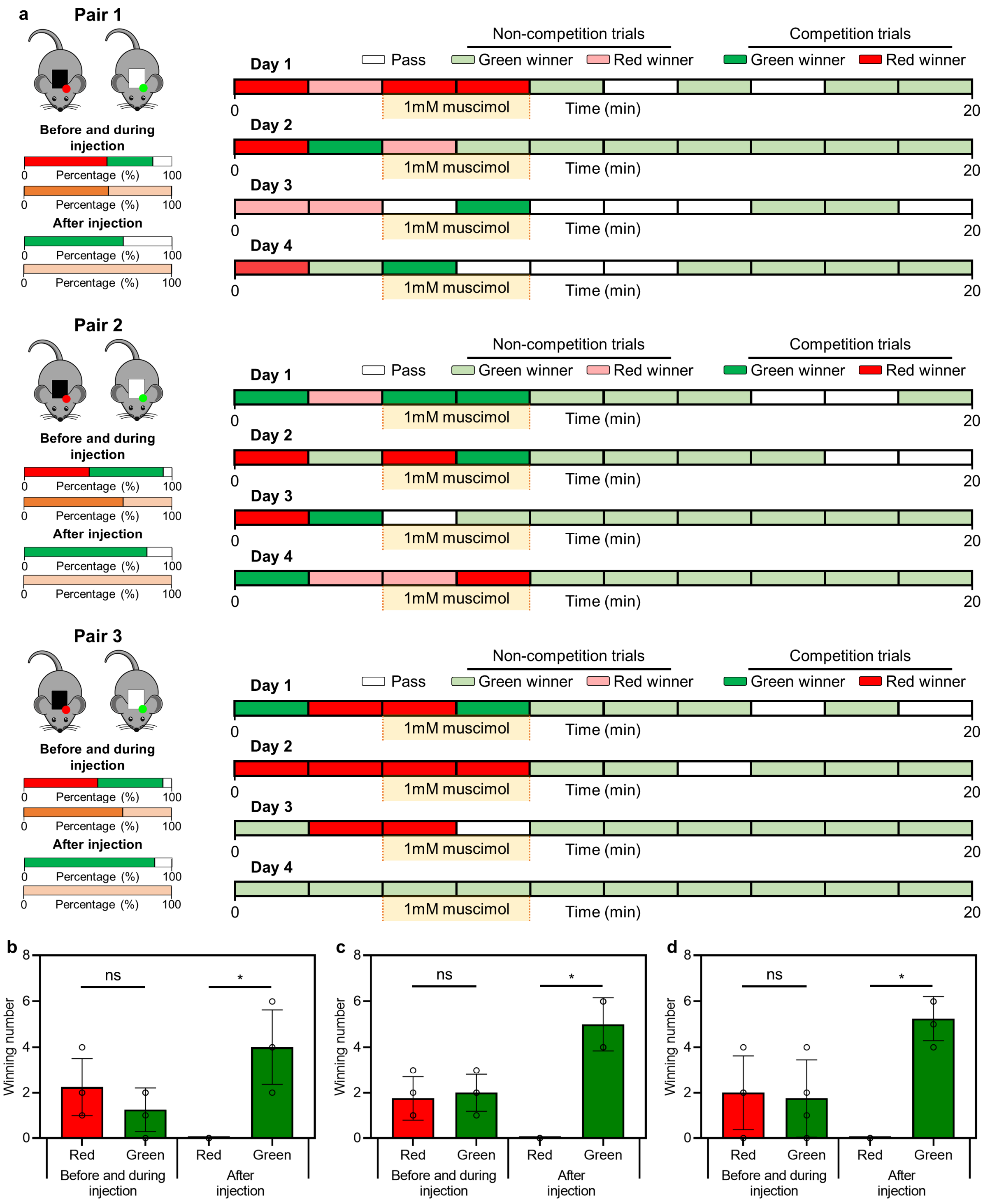

Supplementary Fig. 21: Detailed winning history of each mouse pair throughout the food competition test that included muscimol injection. a Chart showing all dates in each pair. **b-d** Bar graphs showing a comparison of the number of wins between two mice in each session for (**b**) pair 1 (**Before and during injection**: p=0.48570; **After injection**: p=0.02860), (**c**) pair 2 (**Before and during injection**: p=0.91430; **After injection**: p=0.02860), and (**d**) pair 3 (**Before and during injection**:

p=0.97140; **After injection**: p=0.02860). Data are presented as mean values +/− s.d. with individual data points (white circle: n = 4 for all data, n is the number of the test). All statistical analyses were performed by Mann Whitney test. $p<0.05$ was considered significant. * $p< 0.05$. ns: no statistical significance.

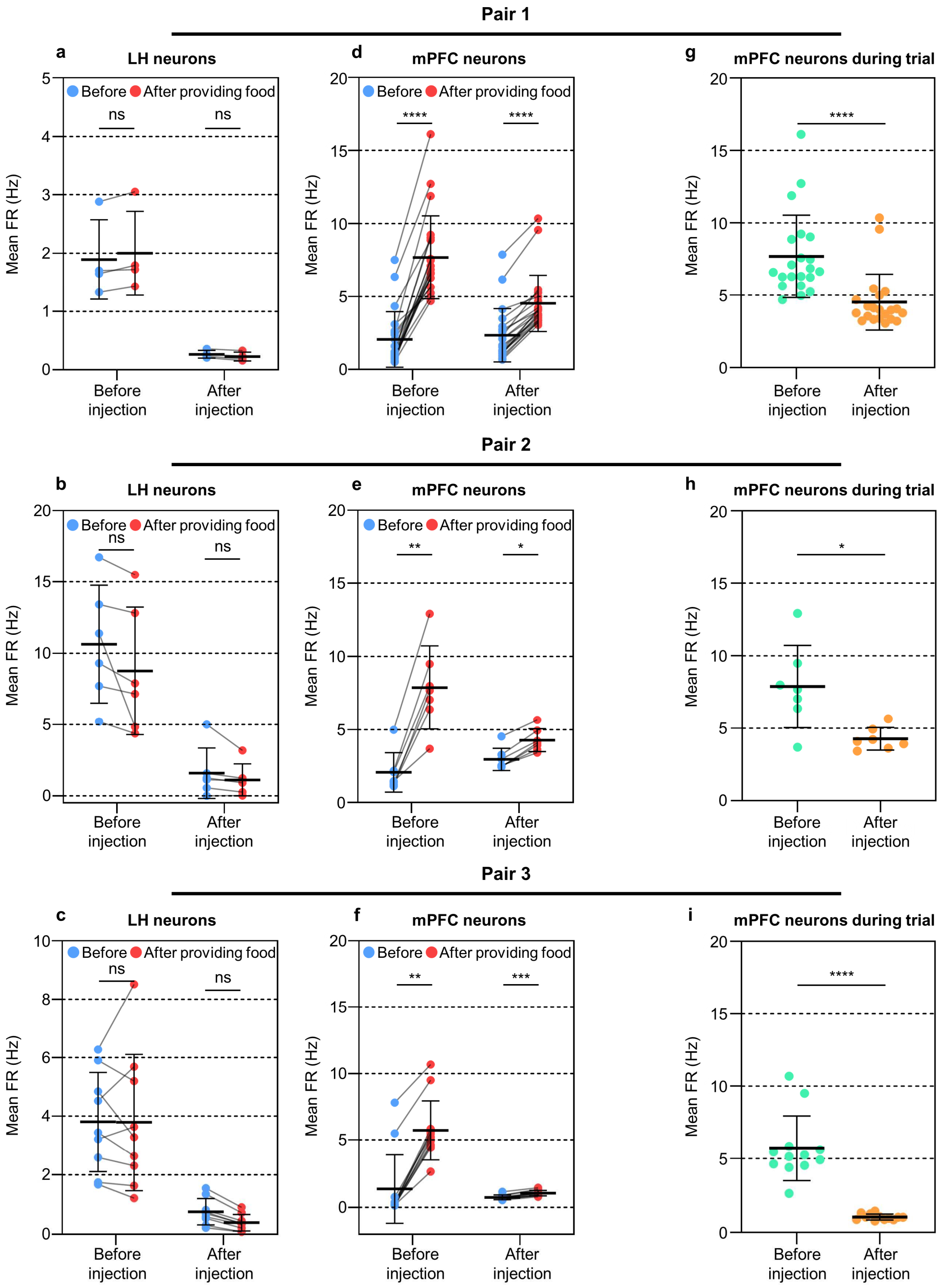

Supplementary Fig. 22: Neural activities of LH and mPFC neurons during food competitions that included muscimol injection. a-c Comparison of mean firing rate in LH neurons of each mouse pair

between before and during trials from before and after muscimol injection (**(a)** before injection: n=4; p=0.48571; after injection: n=4; p=0.40000; **(b)** before injection: n=6; p=0.39394; after injection: n=6; p=0.58874; **(c)** before injection: n=9; p=0.79617; after injection: n=9; p=0.07441). **d-f** Comparison of mean firing rate from mPFC neurons in each mouse pair between before and during trials from before and after muscimol injection (**(d)** before injection: n=21; p<0.00001; after injection: n=21; p=0.00001; **(e)** before injection: n=7; p=0.00117; after injection: n=7; p=0.01107; **(f)** before injection: n=12; p=0.00106; after injection: n=9; p=0.00091). **g-i** Comparison of mean firing rate from mPFC neurons during trials before and after muscimol injection (**(g)** n=21; p<0.00001; **(h)** n=7; p=0.01107; **(i)** before injection: n=12; p<0.00001). Data are presented as mean values +/− s.d. with individual data points. n is the number of neurons. All statistical analyses were performed by Mann Whitney test. p<0.05 was considered significant. * p<0.05, ** p<0.01, *** p< 0.001, **** p<0.0001. ns: no statistical significance.

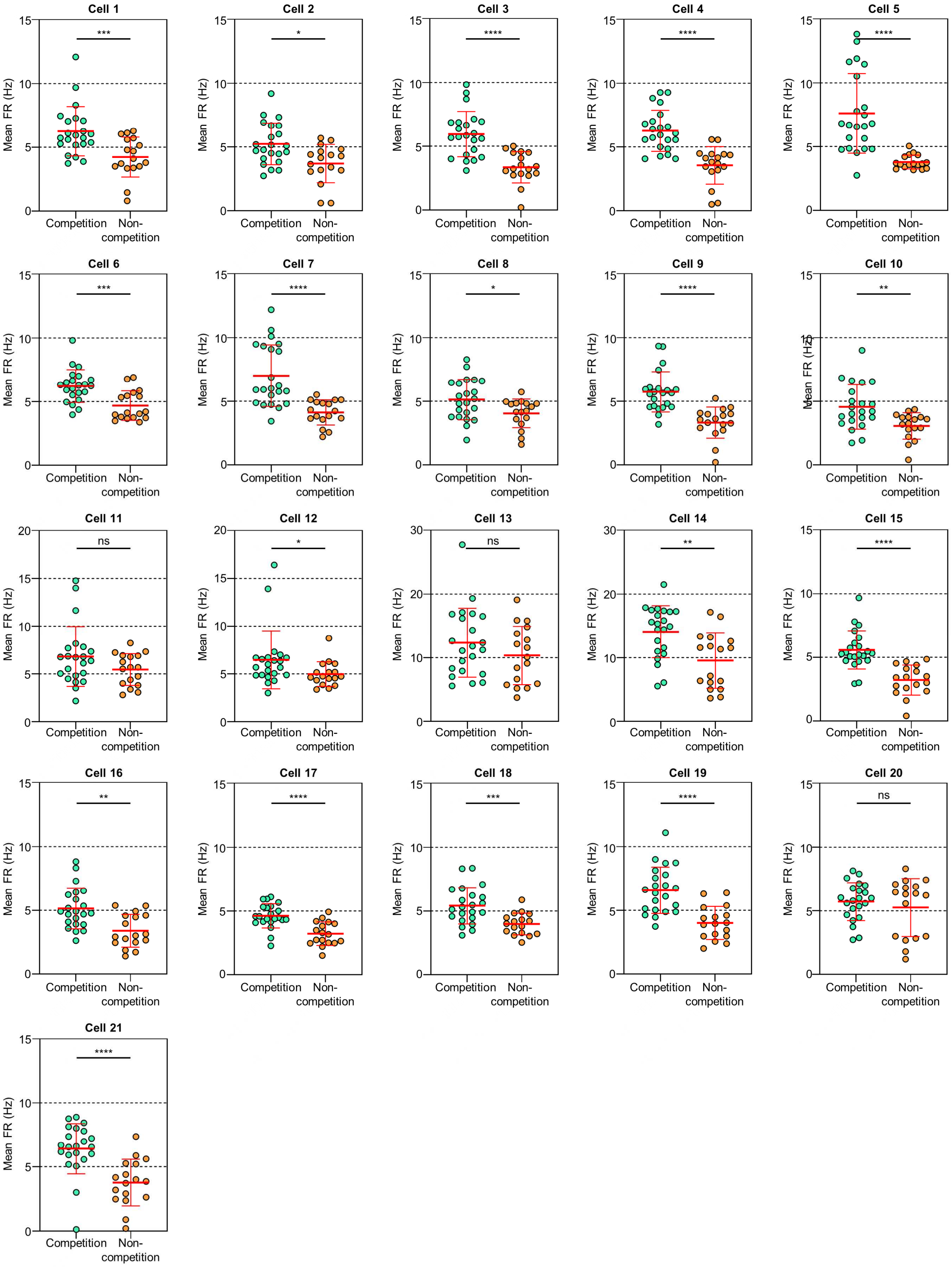

Supplementary Fig. 23: Comparison of neural activities in mPFC between competition and non-competition trials in mouse pair 1 (**cell 1**: p=0.00096; **cell 2**: p=0.01029; **cell 3**: p=0.00001; **cell 4**: p<0.00001; **cell 5**: p<0.00001; **cell 6**: p=0.00043; **cell 7**: p=0.00002; **cell 8**: p=0.04415; **cell 9**:

p<0.00001; **cell 10**: p=0.00448; **cell 11**: p=0.23446; **cell 12**: p=0.01783; **cell 13**: p=0.22073; **cell 14**: p=0.00359; **cell 15**: p<0.00001; **cell 16**: p=0.00142; **cell 17**: p=0.00005; **cell 18**: p=0.00069; **cell 19**: p=0.00001; **cell 20**: p=0.89424; **cell 21**: p=0.00005). Data are presented as mean values +/− s.d. with individual data points (green circle: n=22 for competition, orange circle: n=17 for non-competition; n is the number of trials.). All statistical analyses were performed by Mann Whitney test. p<0.05 was considered significant. * p<0.05, ** p<0.01, *** p< 0.001, **** p<0.0001. ns: no statistical significance.

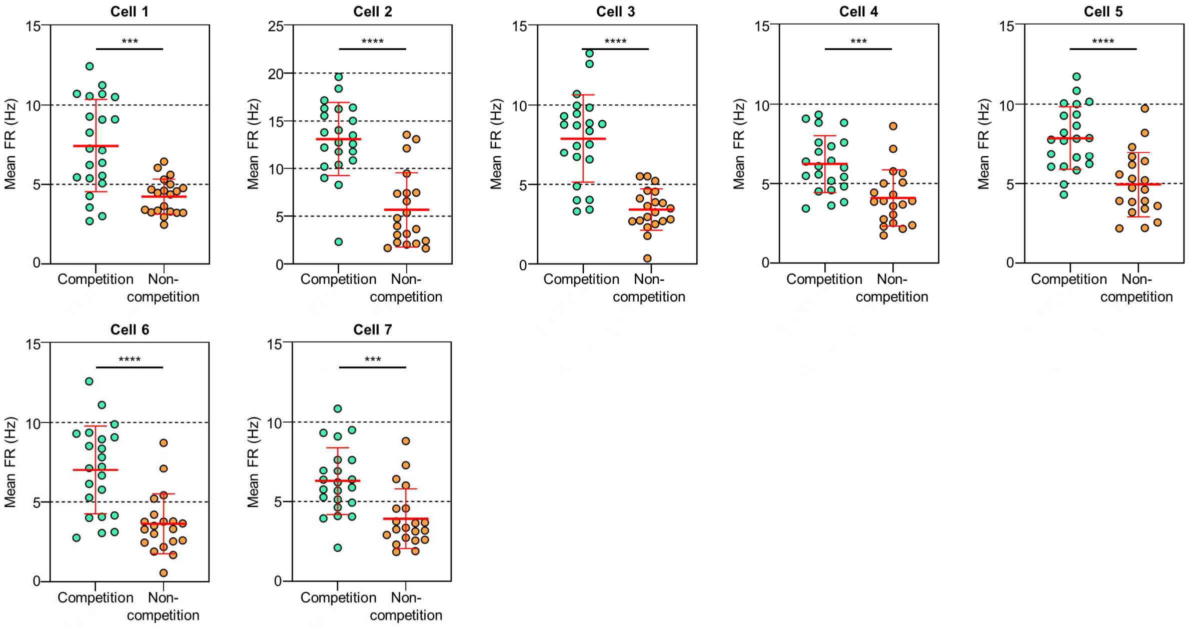

Supplementary Fig. 24: Comparison of neural activities in mPFC between competition and non-competition trials in mouse pair 2 (**cell 1**: p=0.00014; **cell 2**: p<0.00001; **cell 3**: p<0.00001; **cell 4**: p<0.00035; **cell 5**: p=0.00004; **cell 6**: p=0.00005; **cell 7**: p=0.00023). Data are presented as mean values +/− s.d. with individual data points (green circle: n=22 for competition, orange circle: n=20 for non-competition; n is the number of trials.). All statistical analyses were performed by Mann Whitney test. p<0.05 was considered significant. *** p< 0.001, **** p<0.0001.

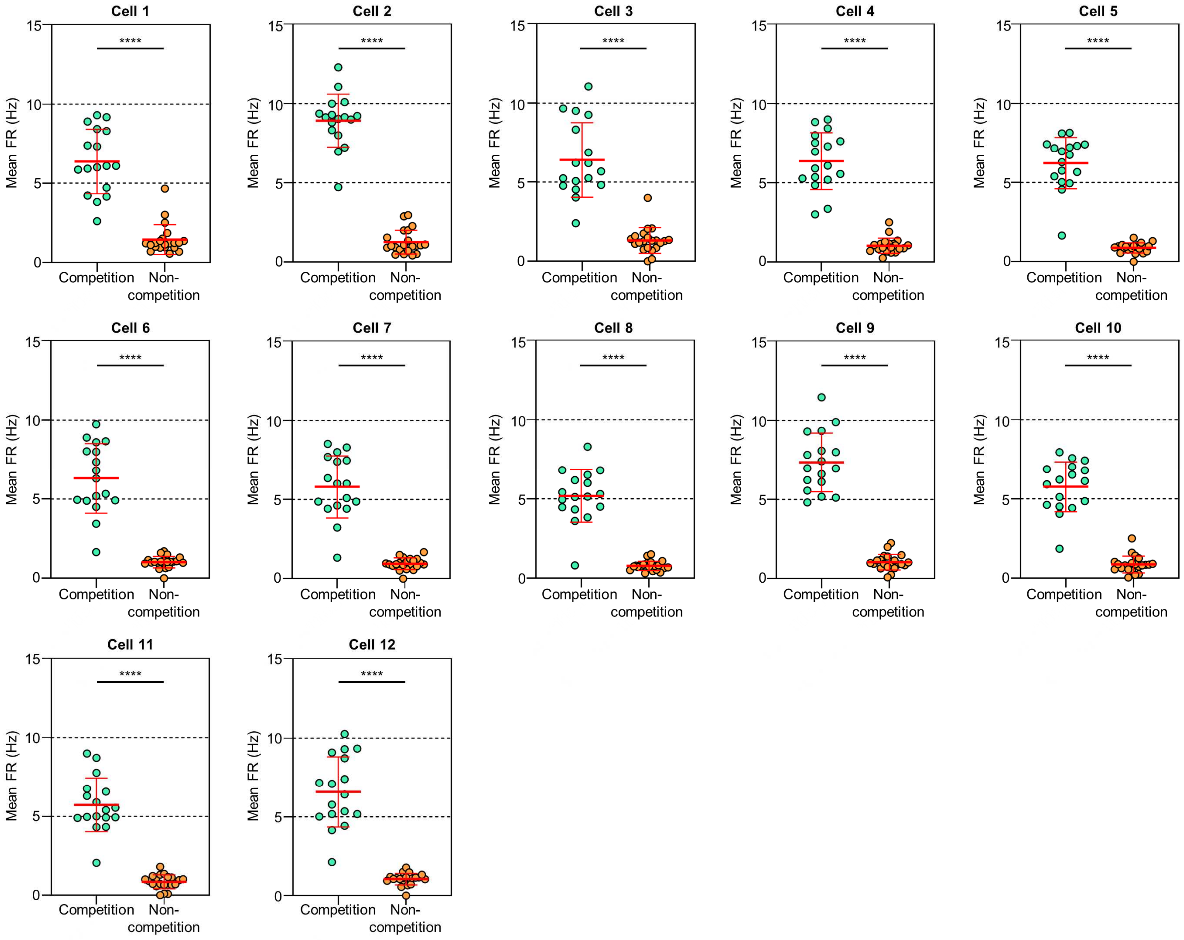

Supplementary Fig. 25: Comparison of neural activities in mPFC between competition and non-competition trials in mouse pair 3 (**cell 1**: p<0.00001; **cell 2**: p<0.00001; **cell 3**: p<0.00001; **cell 4**: p<0.00001; **cell 5**: p<0.00001; **cell 6**: p<0.00001; **cell 7**: p<0.00001; **cell 8**: p<0.00001; **cell 9**: p<0.00001; **cell 10**: p<0.00001; **cell 11**: p<0.00001; **cell 12**: p<0.00001). Data are presented as mean values +/− s.d. with individual data points (green circle: n=17 for competition, orange circle: n=21 for non-competition; n is the number of trials.). All statistical analyses were performed by Mann Whitney test. p<0.05 was considered significant. **** p<0.0001.

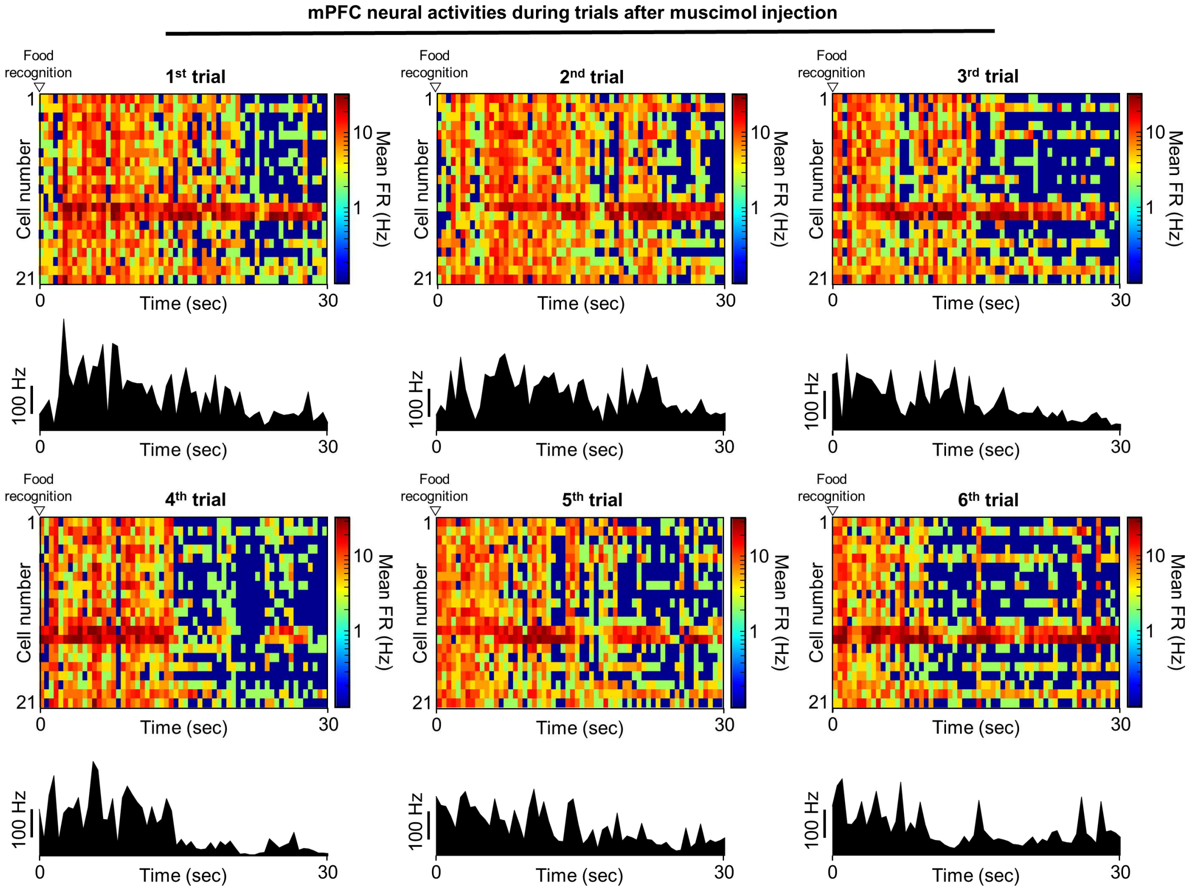

Supplementary Fig. 26: Dynamic changes of mPFC neural activities during each trial after muscimol injection into LH of the opponent: color-mapped raster plots and firing rate histograms showing mPFC neural activities during each trial.

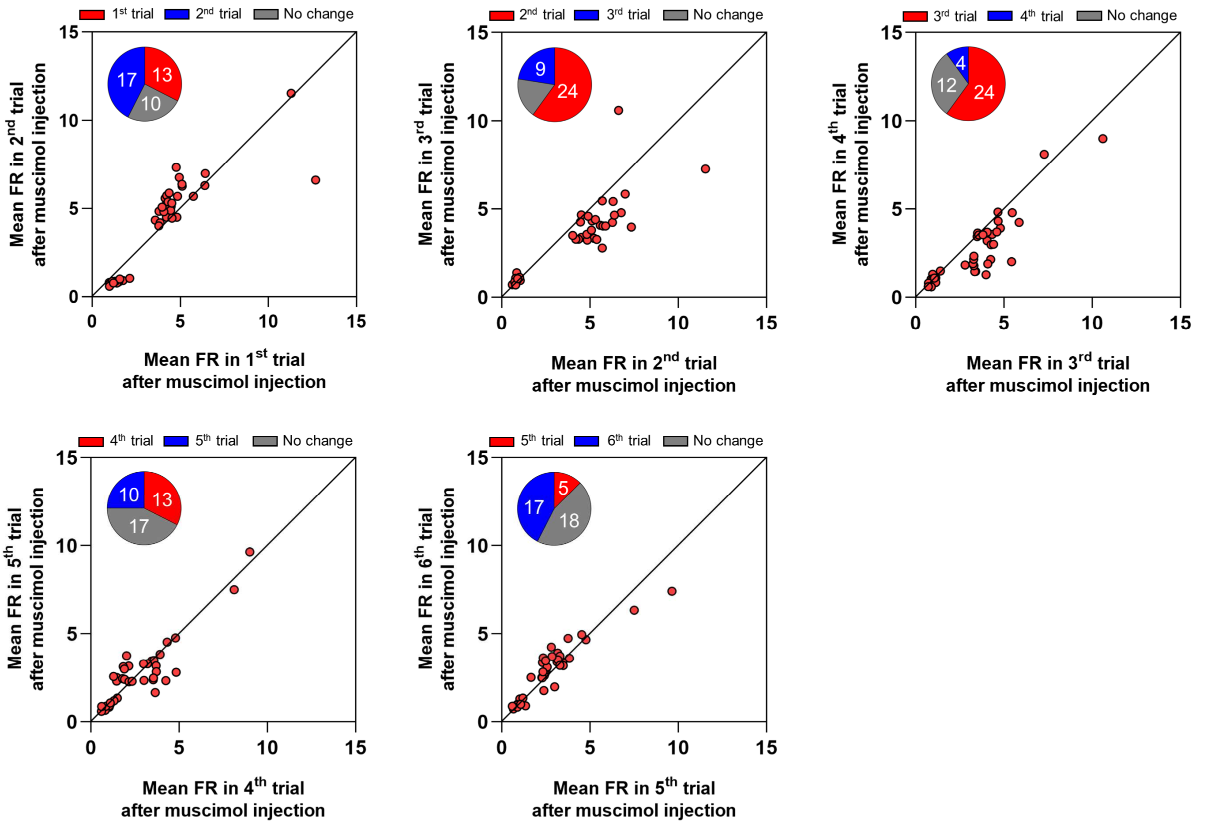

Supplementary Fig. 27: Comparison of mPFC neural activities between trials after muscimol injection into LH of the opponent: scatter plots of the mean firing rate of mPFC neurons during each trial. Colored circles indicate the number of neurons showing significant differences (>10%) in the mean firing rate. In case the difference in the average firing rate between the two trials was within 10%, it was sorted as no change. n is the number of trials (n=40 for all data).

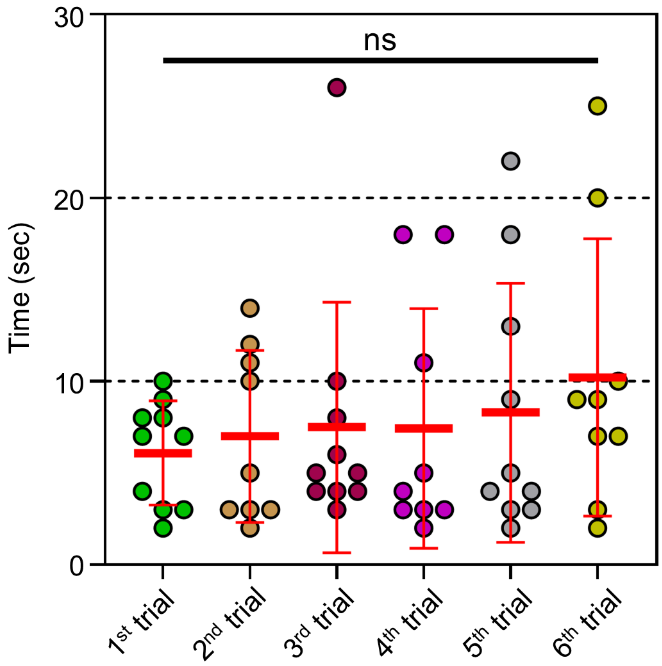

Supplementary Fig. 28: The time for the green mouse to reach the food pellet during successive non-competition trials after muscimol injection. Here, n is the number of non-competition trials and blank in case of pass trial. Data are presented as mean values +/− s.d. with individual data points (colored circles: n = 8 for 1st, 3rd, and 5th trials; n = 7 for 2nd, 4th, and 6th trials). Statistical analysis was performed by one-way mixed-effect ANOVA with Tukey's multiple comparisons test. ns: no statistical significance.

Supplementary Note 1. Estimation of the hydraulic resistance and pressure

We estimated the pressure generated by pump activation based on the measured flow rate and calculated the hydraulic resistance of the probe.

The hydraulic resistance of the probe was calculated using the equation[6] below.

$$R = n \cdot \frac{1}{2\eta} \cdot \frac{(h \cdot w)^3}{l \cdot (2h + 2w)^2} \quad (1)$$

In Supplementary Equation 1, R is hydraulic resistance. n is the number of fluidic channels. η is dynamic viscosity (Pa · s). h, w, and l are the height (μm), width (μm), and length (μm) of the microfluidic channel.

Based on the above equation, we calculated the hydraulic resistance of the probe.

The pressure generated by the pump was estimated using the equation[6] below.

$$p = Q \cdot R \cdot \frac{10^5}{6} \quad (2)$$

In Supplementary Equation 2, p is pressure (Pa). Q is flow-rate (nl·min^{-1}). R is hydraulic resistance.

Based on the calculated hydraulic resistance and the measured flow rate, we estimated the pressure generated by the pump operation.

Supplementary References

1 Dagdeviren, C. et al. Miniaturized neural system for chronic, local intracerebral drug delivery. Sci Transl Med 10, doi:10.1126/scitranslmed.aan2742 (2018).

2 Cobo, A., Sheybani, R., Tu, H. & Meng, E. A Wireless Implantable Micropump for Chronic Drug Infusion Against Cancer. Sens Actuators A Phys 239, 18-25, doi:10.1016/j.sna.2016.01.001 (2016).

3 Zhang, Y. et al. Battery-free, lightweight, injectable microsystem for in vivo wireless pharmacology and optogenetics. Proc Natl Acad Sci U S A 116, 21427-21437, doi:10.1073/pnas.1909850116 (2019)

4 Qazi, R. et al. Wireless optofluidic brain probes for chronic neuropharmacology and photostimulation. Nat Biomed Eng 3, 655-669, doi:10.1038/s41551-019-0432-1 (2019).

5 Shin, H. et al. Interference-free, lightweight wireless neural probe system for investigating brain activity during natural competition. Biosens Bioelectron 195, 113665, doi:10.1016/j.bios.2021.113665 (2022).

6 Lee, H. J. et al. A multichannel neural probe with embedded microfluidic channels for simultaneous in vivo neural recording and drug delivery. Lab Chip 15, 1590–1597 (2015).
