# Supplementary material for: Neural probe system for behavioral neuropharmacology by bi-directional wireless drug delivery and electrophysiology in socially interacting mice
Source: Nat Commun. 2022 Sep 21;13:5521. doi: 10.1038/s41467-022-33296-8 (PMC9492903; doi:10.1038/s41467-022-33296-8)
Supplement: Supplementary file 2 — Description of Additional Supplementary Files [file 41467_2022_33296_MOESM2_ESM.pdf]

## Description of Additional Supplementary Files

**File Name: Supplementary Movie 1**
**Description:** Open field test before and after mounting the neural probe system on the head of the mice for 30 minutes (8x speed).

**File Name: Supplementary Movie 2**
**Description**: Demonstration of the wireless drug delivery using the preliminary configuration of the neural probe integrated with an electrolytic pump (1x speed).

**File Name: Supplementary Movie 3**
**Description:** Surgical procedure (1x speed).

**File Name: Supplementary Movie 4**
**Description:** The connection of the packaged probe to the wireless module in the awake mouse (1x speed).

**File Name: Supplementary Movie 5**
**Description:** Real-time changes of behaviour and neural activities before and after bicuculine (BIC) injection into the substantia nigra (SN) of the freely behaving mouse (4x speed).

**File Name: Supplementary Movie 6**
**Description:** Real-time changes of behaviour and neural activities before and after bicuculine (BIC) injection into the substantia nigra (SN) of the freely behaving mouse (Full length movie) (4x speed).

**File Name: Supplementary Movie 7**
**Description:** Demonstration of repeatable circling behaviour by BIC injection into the SN of the freely behaving mouse for 7 consecutive days (8x speed).

**File Name: Supplementary Movie 8**
**Description:** Demonstration of dose-dependent effect of BIC on circling behaviour by activating the pump differently with 3 cycles and 6 cycles (2x speed).

**File Name: Supplementary Movie 9**
**Description:** Suppression of feeding behaviour by muscimol injection into lateral hypothalamus (LH) of the freely behaving mouse (16x speed).

**File Name: Supplementary Movie 10**
**Description:** Demonstration of social interaction and neural activities in real-time during a food competition test between two mice (4x speed).

**File Name: Supplementary Movie 11**
**Description:** Demonstration of social interaction and neural activities in real-time during a food competition test between two mice while muscimol was injected into the LH of the red mouse (4x speed).

**File Name: Supplementary Movie 12**
**Description:** Real-time neural activities of mPFC for 6 successive trials after muscimol injection into the LH of the red mouse (1x speed).
