# Supplementary material for: Neural probe system for behavioral neuropharmacology by bi-directional wireless drug delivery and electrophysiology in socially interacting mice
Source: Nat Commun. 2022 Sep 21;13:5521. doi: 10.1038/s41467-022-33296-8 (PMC9492903; doi:10.1038/s41467-022-33296-8)
Supplement: Supplementary file 15 — Reporting Summary [file 41467_2022_33296_MOESM15_ESM.pdf]

nature portfolio

Corresponding author(s): Il-Joo Cho

Last updated by author(s): Aug 30, 2022

Reporting Summary

Nature Portfolio wishes to improve the reproducibility of the work that we publish. This form provides structure for consistency and transparency in reporting. For further information on Nature Portfolio policies, see our Editorial Policies and the Editorial Policy Checklist.

Statistics

For all statistical analyses, confirm that the following items are present in the figure legend, table legend, main text, or Methods section.

n/a	Confirmed	
☐	☒	The exact sample size (*n*) for each experimental group/condition, given as a discrete number and unit of measurement
☐	☒	A statement on whether measurements were taken from distinct samples or whether the same sample was measured repeatedly
☐	☒	The statistical test(s) used AND whether they are one- or two-sided<br>*Only common tests should be described solely by name; describe more complex techniques in the Methods section.*
☒	☐	A description of all covariates tested
☒	☐	A description of any assumptions or corrections, such as tests of normality and adjustment for multiple comparisons
☐	☒	A full description of the statistical parameters including central tendency (e.g. means) or other basic estimates (e.g. regression coefficient) AND variation (e.g. standard deviation) or associated estimates of uncertainty (e.g. confidence intervals)
☐	☒	For null hypothesis testing, the test statistic (e.g. *F*, *t*, *r*) with confidence intervals, effect sizes, degrees of freedom and *P* value noted<br>*Give P values as exact values whenever suitable.*
☒	☐	For Bayesian analysis, information on the choice of priors and Markov chain Monte Carlo settings
☐	☒	For hierarchical and complex designs, identification of the appropriate level for tests and full reporting of outcomes
☒	☐	Estimates of effect sizes (e.g. Cohen's *d*, Pearson's *r*), indicating how they were calculated

Our web collection on statistics for biologists contains articles on many of the points above.

Software and code

Policy information about availability of computer code

Data collection	Custom recording software, Tera Term 4.91 (Tera Term Project, open source software), Windows10 Camera (Microsofts), Matlab2016a (Matlab), NanoZ 1.4 (Neuralynx), UTCK 3.1 (Korea Research Institute of Standards and Science), IAR Embedded Workbench for MSP430 version 7.12.1 (IAR Systems)
Data analysis	Matlab2016a (Matlab) was used to extract recording data and to display the trajectory plots.<br>Python 3.7.4 (Python software foundation) was used to detect the neural spikes in recorded data and to display the trajectory plots.<br>The open source packages spike_sorting, which are available at https://github.com/akcarsten/spike_sorting/.idTracker 2.1 (https://www.idtracker.es/) and Optimouse 3.0 (https://github.com/yorambenshaul/optimouse) was used to track the path of movement of the mice.<br>Also, the custom codes applied in the wireless communication module and used for data extraction, and custom software for neural signal recording are freely available at https://github.com/HyogeunShin/Wireless-System_NatComm22 (https://doi.org/10.5281/zenodo.7032078).<br>GraphPad Prism 7.04 (GraphPad Prism) was used to display the graphs and to perform statistical analysis.

For manuscripts utilizing custom algorithms or software that are central to the research but not yet described in published literature, software must be made available to editors and reviewers. We strongly encourage code deposition in a community repository (e.g. GitHub). See the Nature Portfolio guidelines for submitting code & software for further information.

Data

Policy information about availability of data

All manuscripts must include a data availability statement. This statement should provide the following information, where applicable:

- Accession codes, unique identifiers, or web links for publicly available datasets
- A description of any restrictions on data availability
- For clinical datasets or third party data, please ensure that the statement adheres to our policy

The authors declare that all data supporting the findings of this study are available within the article and its supplementary information files. Source data are provided with this paper.

Field-specific reporting

Please select the one below that is the best fit for your research. If you are not sure, read the appropriate sections before making your selection.

- [x] Life sciences
- [ ] Behavioural & social sciences
- [ ] Ecological, evolutionary & environmental sciences

For a reference copy of the document with all sections, see nature.com/documents/nr-reporting-summary-flat.pdf

Life sciences study design

All studies must disclose on these points even when the disclosure is negative.

Sample size	Sample size is indicated in th figure legend for each experiment. We did not use a computational method to determine sample size. We chose based on previous experience with signal and behaviour analysis from behaving animals and determined sample size to be appropriate based on the consistency and magnitude of measurable differences in experiments.<br><br>Reference: Shin, H. et al. Interference-free, lightweight wireless neural probe system for investigating brain activity during natural competition. Biosens Bioelectron 195, 113665, doi:10.1016/j.bios.2021.113665 (2022).
Data exclusions	No data were excluded.
Replication	All experiments were repeated independently at least three times with similar result. All replicates were successfully performed.
Randomization	Samples were randomly assigned to experiments.
Blinding	The investigators were not blinded to group allocation during data collection. Blinding was not necessary because analysis for signal data was performed automatically using MATLAB and Python, with the same code run on each experiments. Also, behavioral analysis was performed by researchers who were not directly involved in the experiment.

Reporting for specific materials, systems and methods

We require information from authors about some types of materials, experimental systems and methods used in many studies. Here, indicate whether each material, system or method listed is relevant to your study. If you are not sure if a list item applies to your research, read the appropriate section before selecting a response.

Materials & experimental systems

n/a	Involved in the study	
x		Antibodies
x		Eukaryotic cell lines
x		Palaeontology and archaeology
	x	Animals and other organisms
x		Human research participants
x		Clinical data
x		Dual use research of concern

Methods

n/a	Involved in the study	
x		ChIP-seq
x		Flow cytometry
x		MRI-based neuroimaging

Animals and other organisms

Policy information about studies involving animals; ARRIVE guidelines recommended for reporting animal research

Laboratory animals	Adult male C57BL/6 mice (10 weeks old) were used for the in vivo experiments. Five or six mice were housed in a cage that had a 12:12 light-dark cycle. The temperature and humidity of the animal facility were maintained at 22±2°C and 50±5%.

Wild animals	No wild animals were used in the study.
Field-collected samples	No field collected samples were used in the study.
Ethics oversight	All of the procedures, including the use of animals, were approved by the Korea Institute of Science and Technology (KIST) in Seoul, Korea, and the procedures were conducted in accordance with the ethical standards stated in the Animal Care and Use Guidelines of KIST.

Note that full information on the approval of the study protocol must also be provided in the manuscript.
